# The RNA Complement of Outer Membrane Vesicles From *Salmonella enterica* Serovar Typhimurium Under Distinct Culture Conditions

**DOI:** 10.3389/fmicb.2018.02015

**Published:** 2018-08-30

**Authors:** Antoine Malabirade, Janine Habier, Anna Heintz-Buschart, Patrick May, Julien Godet, Rashi Halder, Alton Etheridge, David Galas, Paul Wilmes, Joëlle V. Fritz

**Affiliations:** ^1^Luxembourg Centre for Systems Biomedicine, University of Luxembourg, Esch-sur-Alzette, Luxembourg; ^2^UMR CNRS 7021, Laboratoire de BioImagerie et Pathologies, Université de Strasbourg, Strasbourg, France; ^3^Pacific Northwest Research Institute, Seattle, WA, United States

**Keywords:** outer membrane vesicle (OMV), RNA export, *Salmonella enterica* pathogenicity, host–pathogen interaction, SPI-1, SPI-2, virulence, sRNA

## Abstract

Bacterial outer membrane vesicles (OMVs), as well as OMV-associated small RNAs, have been demonstrated to play a role in host–pathogen interactions. The presence of larger RNA transcripts in OMVs has been less studied and their potential role in host–pathogen interactions remains largely unknown. Here we analyze RNA from OMVs secreted by *Salmonella enterica* serovar Typhimurium (*S.* Typhimurium) cultured under different conditions, which mimic host–pathogen interactions. *S.* Typhimurium was grown to exponential and stationary growth phases in minimal growth control medium (phosphate-carbon-nitrogen, PCN), as well as in acidic and phosphate-depleted PCN, comparable to the macrophage environment and inducing therefore the expression of *Salmonella* pathogenicity island 2 (SPI-2) genes. Moreover, *Salmonella* pathogenicity island 1 (SPI-1), which is required for virulence during the intestinal phase of infection, was induced by culturing *S.* Typhimurium to the stationary phase in Lysogeny Broth (LB). For each condition, we identified OMV-associated RNAs that are enriched in the extracellular environment relative to the intracellular space. All RNA classes could be observed, but a vast majority of rRNA was exported in all conditions in variable proportions with a notable decrease in LB SPI-1 inducing media. Several mRNAs and ncRNAs were specifically enriched in/on OMVs dependent on the growth conditions. Important to note is that some RNAs showed identical read coverage profiles intracellularly and extracellularly, whereas distinct coverage patterns were observed for other transcripts, suggesting a specific processing or degradation. Moreover, PCR experiments confirmed that distinct RNAs were present in or on OMVs as full-length transcripts (*IsrB-1/2; IsrA*; *ffs*; *SsrS*; *CsrC*; *pSLT035*; *10Sa*; *rnpB*; *STM0277*; *sseB*; *STM0972*; *STM2606*), whereas others seemed to be rather present in a processed or degraded form. Finally, we show by a digestion protection assay that OMVs are able to prevent enzymatic degradation of given full-length transcripts (*SsrS*, *CsrC*, *10Sa*, and *rnpB)*. In summary, we show that OMV-associated RNA is clearly different in distinct culture conditions and that at least a fraction of the extracellular RNA is associated as a full-length transcripts with OMVs, indicating that some RNAs are protected by OMVs and thereby leaving open the possibility that those might be functionally active.

## Introduction

The secretion of biomolecules into the environment is a common feature of living cells, as organisms can communicate, eliminate undesired products, protect themselves or mediate pathogenicity by the means of export. A wide variety of secretion pathways exist, such as direct export, encapsulation or vesicle formation. Gram-negative bacteria possess several of them, with exports of molecules through the passage across their membranes and cell walls or by the release of outer membrane vesicles (OMVs). OMVs are 50–250 nm spherical particles derived from the outer membrane of Gram-negative bacteria, containing a wide range of molecules. Their release is a phenomenon observed constitutively in many Gram-negative bacteria, including *Salmonella* (Schwechheimer and Kuehn, 2015). OMV formation is still poorly understood, even though recent advances have highlighted the importance of the LPS synthesis pathway (Schwechheimer et al., 2013; Bonnington and Kuehn, 2016; Elhenawy et al., 2016). Environmental conditions such as oxygen or iron availability, growth phase and media composition influence vesicle biogenesis (Orench-Rivera and Kuehn, 2016). Therefore, to shed light on the identification of OMV-associated molecules it becomes essential to study the content of OMVs originating from different culture conditions.

Outer membrane vesicles’ physiological roles are broad, ranging from functions such as nutrient acquisition, sharing of resistance, stress response or biofilm reinforcement, to more offensive roles like toxin concentration, delivery of virulence factors or modulation of host immune defenses (Manning and Kuehn, 2013; Jan, 2017). Their role and importance in intra- or interspecies communication and in pathogenicity is of growing interest (Schwechheimer and Kuehn, 2015; Jan, 2017; Lynch and Alegado, 2017). Whereas the type III, type IV, and type VI secretion systems imply direct contact between bacteria and their host to allow the release of molecules into the host cytosol, the release of OMVs provides a way to transfer bacterial cargo without being in close contact with the host and thus OMVs can act as long distance carriers (Jan, 2017; Tashiro et al., 2017). In this context, the classification of OMVs as a type 0 secretion system has been proposed recently (Guerrero-Mandujano et al., 2017). OMV delivery to their target occurs by different means, such as membrane fusion with the host or a variety of endocytosis mechanisms (O’Donoghue and Krachler, 2016). OMV-associated biomolecules mainly consist of the periplasmic content, but also contain outer membrane proteins such as OmpA (Bai et al., 2014) as well as a variety of lipids, nucleic acids, proteins and metabolites from the cytoplasm (Jan, 2017).

Over the past few years, several studies on mammalian cells suggest that the horizontal transfer of secreted RNAs occurs between cells and this points to a novel role for these molecules in intercellular and interspecies communication [summarized in the following review: (Fritz et al., 2016)]. So far, intercellular transfer of extracellular RNA molecules has not only been shown in mammals but also in fungi and in bacteria. Indeed, a plant-residing fungal pathogen has been shown to be able to transfer specific RNA effectors into its host cells to suppress host immunity (Weiberg et al., 2013). Moreover, extracellular RNA secreted by *Listeria* spp. are key components for developing immunity against the bacterial infection (Abdullah et al., 2012) and extracellular RNA fragments of *Mycobacterium tuberculosis* have been shown to be able to induce early apoptosis in human monocytes (Obregón-Henao et al., 2012). Finally, an OMV-associated small RNA has been demonstrated to participate in *Pseudomonas* pathogenicity by reducing the host immune response (Koeppen et al., 2016). Thus, for the moment only a few examples of interspecies communication *via* extracellular RNA are known, but the fact that such pathways have been identified indicates that communication *via* extracellular RNAs seem to be species-conserved and could be a widespread and important mechanism.

Generally, it is assumed that by using a microvesicle-dependent active trafficking system, secreted RNAs can be delivered into recipient cells where they function analogous to endogenous RNAs, and take part in regulating multiple target genes or signaling events in intra- and interspecies communication [reviewed in: (Chen et al., 2012)]. Interestingly, OMVs contain a wide range of RNAs including ribosomal RNA (rRNA), transfer RNA (tRNA), messenger RNA (mRNA), and small non-coding RNA (sRNA) (Ghosal et al., 2015; Ho et al., 2015; Sjöström et al., 2015; Blenkiron et al., 2016; Choi et al., 2017; Soares et al., 2017; Dauros-Singorenko et al., 2018), which indicates that OMVs are important to study in the context of extracellular RNA communication.

To study OMV-associated RNA in the context of host–pathogen interaction, *S.* Typhimurium seems to be a good model as *Salmonella* does secrete OMVs (Schwechheimer and Kuehn, 2015) and it has been shown that some *in vitro* culture conditions are able to mimic different steps of the *Salmonella* life cycle (Ibarra et al., 2010; Kröger et al., 2013). *Salmonella* encounters different environments during its life cycle and exhibits different behaviors in each infection step. Thereby, one distinguishes between the invasive state where bacteria are extracellular, in the gut, generally flagellated, and the intracellular state when *Salmonella* has entered the epithelium and then propagates to macrophages or dendritic cells to replicate (Fabrega and Vila, 2013; LaRock et al., 2015). Depending on the environment, different expression profiles are generated by a complex regulatory network of virulence factors, encoded in several of the so-called *Salmonella* Pathogenicity Islands (SPI), on the chromosome and on the pSLT virulence plasmid (Yoon et al., 2009; Hébrard et al., 2011; Fabrega and Vila, 2013). SPI-1 genes, which are triggered when *Salmonella* encounters epithelial cells, can be activated when *Salmonella* is grown to a high optical density (OD) within Lysogeny Broth (LB) (Ibarra et al., 2010; Kröger et al., 2013). Similarly, the infection of macrophages by *Salmonella*, where SPI-2 genes are required, can be partially reproduced by a specific minimal medium with low pH and low phosphate availability (Beuzón et al., 1999; Srikumar et al., 2015). *Salmonella* RNA expression during host–pathogen interactions is a dynamic process, with significant modulation as the infection progresses (Hautefort et al., 2008; Ortega et al., 2012; Kröger et al., 2013; Avital et al., 2017).

In this study, we aimed to characterize the RNA cargo of *Salmonella* Typhimurium OMVs through OMV isolation and RNA sequencing, in different culture conditions representative of some of the environments encountered by the bacteria during the infection process.

## Materials and Methods

### Strain and Culture Conditions

*Salmonella enterica* subsp. *enterica* serovar Typhimurium LT2 (henceforth referred to as *S.* Typhimurium) preculture triplicates were grown overnight in control (PCN), SPI-2 inducing (SPI-2ind) or SPI-1 inducing (SPI-1ind) conditions at 37°C and 160 Rotations Per Minute (RPM). Control conditions consisted of PCN buffer (pH 7.4, 80 mM MOPS, 50 mM NaCl, 25 mM K_2_HPO_4_, 22.2 mM glucose, 15 mM NH_4_Cl, 4 mM Tricine, 1 mM MgSO_4_, 376 μM K_2_SO_4_, 100 μM FeCl_3_, 10 μM CaCl_2_, 800 nM MnCl_2_, 300 nM CoCl_2_, 100 nM CuSo_4_, 10 nM Na_2_MoO_4_, 10 nM Na_2_SeO_3_, 4 nM boric acid, and 1 nm ZnSO_4_). SPI-2ind conditions (inducing *Salmonella* Pathogenicity Island 2) consisted of PCN buffer with lower pH (5.8, 80 mM MES replacing MOPS) and low inorganic phosphate content (0.4 mM K_2_HPO_4_) (Löber et al., 2006; Kröger et al., 2013). SPI-1ind conditions (inducing *Salmonella* Pathogenicity Island 1) consisted of LB supplemented to 10 g.l^-1^ NaCl [according to Miller, (Miller, 1972)] grown until early stationary phase (Kröger et al., 2013). 1.5 l cultures in the same respective conditions were inoculated the next day and grown until the desired growth phase (i.e., log phase with an OD_600nm_ of 0.4 for Control Low OD and SPI-2ind Low OD; and stationary phase with an OD_600nm_ 0.8 for SPI-2ind High OD or 1.6 for Control High OD and SPI-1ind). At this step, a viability check was included for all the different culture conditions to ensure that purified OMVs would not be contaminated with membrane debris originating from lysed cells (LIVE/DEAD BacLight Bacterial Viability Kit, Invitrogen). **Supplementary Figure S1** shows that hardly any dead bacteria were apparent for any of the culture conditions tested within this study.

### OMV Purification and RNA Extraction

Outer membrane vesicles extraction was performed as described previously (Habier et al., 2018). Briefly, bacteria, grown as described above, were centrifuged (4700 *g*, 10 min, 4°C) and the pellet was stored at -80°C in order to be able to extract intracellular RNA at a later time point. The intracellular pellet was conserved without any RNA preservation agent. 10^9^ cells were used for extraction of intracellular RNAs. The whole 1,5 l supernatant was filtered using a 0.22 μm filter before being concentrated by ultrafiltration using tangential flow (Quixstand Benchtop System with 100-kDa hollow-fiber membrane). The collected OMVs-enriched liquid was then ultracentrifuged using a precooled Beckman ultracentrifuge with a 90Ti rotor during 3 h at 150,000 *g* and 4°C to obtain crude OMVs (before density gradient separation). After resuspension of the crude OMVs in 500 μl of buffer (50 mM Hepes, 150 mM NaCl and pH 6.8), pure OMVs were isolated by iodixanol gradient (Optiprep, Sigma) using a precooled Beckman ultracentrifuge with a swinging bucket rotor (SW40 Ti) during 16 h at 100,000 *g* and 4°C. Continuous gradients were built with a bottom-up approach, loading vesicles in the 2 ml bottom fraction containing 45% w/v iodixanol, which was overlaid successively by 2 ml of 40, 35, 30, 25, and 20% w/v iodixanol fractions. After overnight (16 h) ultracentrifugation, 12 fractions of 1 ml were collected from the top low-density fractions to the bottom high-density fractions. The OMV-containing fractions were identified by subjecting 12.5 μl of each of the 12 isolated iodixanol fractions to SDS-PAGE followed by Western-Blot with an anti-OmpA IgG (Biorbyt) (for an example see **Supplementary Figure S2A**). Once identified, the remaining 987,5 μl of each OMV-containing fraction (positive for OmpA) was pooled and subjected to Trichloroacetic Acid precipitation (final concentration 10% w/v) followed by centrifugation during 30 min at 17136 *g* and 4°C. The pellet was resuspended in 450 μl ice-cold 80% acetone before a further centrifugation step for 30 min at 17136 *g* and 4°C. After acetone evaporation, the pellet was resuspended in 100 μl of buffer (50 mM Hepes, 150 mM NaCl, pH 6.8). Vesicles were treated with freshly prepared lysozyme (from chicken-egg white, Sigma-Aldrich, final concentration 1 μg.ml^-1^), even though this step might be superfluous as lysozyme digest sugars, but we aimed treating intracellular and extracellular samples as similarly as possible in order to be able to compare the different samples later on. RNA extraction was then performed with the All-in-One Purification Kit (Norgen), allowing the separation of the RNA within a small RNA (<200 nt) and a large RNA (>200 nt) fraction. The “large RNA fraction” (>200 nt) has been used for RNA-Seq, even though our data clearly indicates that all small RNAs (except tRNAs) can be resolved by RNA-Seq within the “large RNA fraction” (**Figures 3**, **11**). OMV-associated RNA was further concentrated using a Zymo Research RNA clean up and concentrator kit (including On-column DNaseI treatment) up to a volume of 6 μl (corresponding to 1.5 l of original culture). The extraction of intracellular RNA was also performed with the All-in-One Purification Kit (Norgen), followed by a DNaseI treatment. 1 μl of RNA was quality-checked with an Agilent 2100 Bioanalyzer. Detected RNAs were longer than 25 nucleotides in length (see **Supplementary Figure S3**).

### OMV Characterization

#### Transmission Electron Microscopy

##### Sample preparation

The presence of OMVs after density gradient separation was confirmed by Transmission Electron Microscopy (TEM). For this purpose, the SPI-1ind condition has been used. OMV purification was performed as stated before but each of the 12 obtained iodixanol fractions obtained after gradient ultracentrifugation was fixed with 4% w/v paraformaldehyde during 10 min. OMVs were then rinsed with 8 ml of buffer (50 mM Hepes, 150 mM NaCl and pH 6.8) and pelleted using a precooled Beckman ultracentrifuge with a 90Ti rotor during 3 h at 150,000 *g* at 4°C. The pellet was finally resuspended in 100 μl of the same buffer. A sample of the crude OMV preparation was also observed by TEM.

A 5 μl drop of each sample was deposited on a glow-discharged carbon-coated Electron Microscopy (EM) copper grid (400 mesh square grid, EMS). After 5 min of application, the excess of sample was blotted out using a Whatman filter paper and then the grid was washed with water three times to remove any buffer salts, which would react with uranyl acetate used for negative staining. To perform negative staining, 5 μl of uranyl acetate solution (2%) was applied on the grids. After 30 s of incubation, the excess of uranyl acetate was blotted out and then the grids were kept in a dry, dark, dust-free environment until observation with the electron microscope.

##### Sample observation

The EM grids were mounted on to a room temperature-equilibrated holder and subsequently introduced into a FEI Tecnai 20 transmission electron microscope (FEI Eindhoven Holland). Images (2048 pixels × 2048 pixels) were acquired using a US1000 camera (Gatan) at 50000× (nominal magnification, the corresponding pixel size was 0.21 nm). OMVs were found in gradient fractions where OmpA was detected by Western-blot, demonstrating that the OmpA screening is a suitable method for identifying OMV-enriched iodixanol gradient fractions (see **Supplementary Figure S2**).

#### Fluorescence Correlation Spectroscopy

In order to have an estimate of the amount of OMVs secreted by *S.* Typhimurium, OMVs were concentrated from a 0.75 l culture in SPI-1ind condition by tangential flow as explained previously. Vesicles were subsequently fixed with 4% w/v paraformaldehyde during 10 min, rinsed with 8 ml of buffer (50 mM Hepes, 150 mM NaCl and pH 6.8) and pelleted using a precooled Beckman ultracentrifuge with a 90Ti rotor during 3 h at 150 000 *g* at 4°C. The pellet was finally resuspended in 500 μl of the same buffer. A portion of the culture was plated on LB agar plates to determine CFU.ml^-1^ (Colony Forming Units). Quantification was not performed on samples subjected to density gradient separation, as it is not possible to obtain the dilution factor of this technique, which is needed to obtain OMVs/CFU in the original culture.

Quantification of OMVs was achieved by Fluorescence Correlation Spectroscopy (FCS). OMVs were stained with 100 nM of Nile Red lipophilic fluorescent dye (Sigma-Aldrich). Measurements were performed on a two-photon platform, as previously described (Didier et al., 2009). Briefly, two photon excitation is provided by an InSight DeepSee Laser (Spectra Physics). Pulses of about 100 fs were produced at a wavelength of 830 nm. FCS measurements were performed on an Olympus- I×70 inverted microscope. The laser beam was focused into the sample by a water-immersion objective (60× 1.2 NA Olympus) generating a diffraction-limited focal spot of about 0.6 μm in diameter (∼0.3 μm^3^). The measurements were carried out in a 8 Well Lab-Tek II cover glass system. The sample fluorescence was collected through the same objective and directed by a dichroic mirror (COWL 750 nm; Coherent) to the lateral output of the microscope. After rejection of residual IR light by a short pass filter (E700SP, Chroma), the emitted photons were focused by a 200 mm achromatic lens on the 64 μm core of a multimode optical fiber coupled to an avalanche photodiode (SPCM-AQR-14-FC, EG&G). The detected signal was correlated online by an ALV-5000E correlator (ALV, Germany) to generate autocorrelation curves *G*(τ). The analysis of *G*(τ) can provide information about the underlying mechanisms responsible for the intensity fluctuations such as number and diffusion of the particles. For an ideal case of freely diffusing monodisperse fluorescent particles undergoing a triplet-like blinking process in a Gaussian excitation volume, the correlation function, *G*(τ), calculated from the fluorescence fluctuations can be fitted according to:

$$G(\tau )=\frac{1}{N}{\left(1+\frac{\tau }{\tau_{d}}\right)}^{-1}{\left(1+\frac{1}{S^{2}}\frac{\tau }{\tau_{d}}\right)}^{-0.5}\left(1+\frac{f_{t}}{1-f_{t}}e^{-\frac{\tau }{\tau_{t}}}\right)$$

where τ*_d_* is the apparent diffusion time (a parameter that is inversely related to the diffusion constant of the molecule), *N* is the mean number of molecules within the excitation volume, *S* is the ratio between the axial and lateral radii of the sample volume (*S* ∼ 5), *ft* is the mean fraction of fluorophores in their dark state and τ*_t_* is the triplet state lifetime. Using carboxytetramethylrhodamine (TMR) in water as a reference (*D_TMR_* = 421 μm^2^.s^-1^), the diffusion coefficient, *D_exp_*, of the labeled OMVs was calculated by:

$$D_{\exp}=D_{TMR}\times \frac{\tau_{d}\,\;\left(TMR\right)}{\tau_{d}\;(sample)}$$

where τ*_d_* (*TMR*) and τ*_d_* (*sample*) are the measured correlation times for TMR and the sample, respectively. Typical data recording times were 10 min. Quantification results gave us an estimate of 8.51 OMVs/CFU in SPI-1ind condition (see **Supplementary Figure S4**).

### RNA Sequencing

RNA sequencing was performed for Low OD and High OD samples in two distinct sequencing facilities, with minor changes indicated below. 1 μg of intracellular RNA was subjected to rRNA depletion using the Ribo-Zero Magnetic Kit (Gram-negative Bacteria) (Illumina, MRZB 12424), to obtain a sufficient sequencing depth. For better insight in the composition of OMV-associated RNAs, the rRNA depletion step was omitted for OMV libraries. RNA-Sequencing libraries were prepared using the TruSeq Stranded mRNA LT kit (Illumina, used for High OD samples) or the KAPA stranded RNA kit (Kapa Biosystems, used for Low OD samples) without mRNA pull down steps according to the manufacturer’s protocol. The insert size and the quality of libraries were checked with an Agilent 2100 Bioanalyzer and quantified using the Qubit dsDNA HS assay kit. Libraries were diluted to 4 nM each, pooled, denatured and sequenced 2 × 80 (Low OD) or 2 × 75 (High OD) cycles on a NextSeq500 (Illumina) according to the manufacturer’s instructions. For each experimental condition, three biological replicates were sequenced. The raw sequence libraries are deposited in the BioProject database under the experiment accession number PRJNA438234, available at https://www.ncbi.nlm.nih.gov/bioproject/438234.

### RNAseq Data Treatment and Analysis

#### RNA Sequencing Annotation

Raw RNA 75 nt or 80 nt paired-end sequencing reads from each condition were first trimmed and quality filtered using the *trim-fastq.pl* script from *PoPoolation* package with the following parameters – quality-threshold 20 – fastq-type sanger – min-length 35 (Kofler et al., 2011). The *Salmonella* genome and plasmid sequences and annotations (in GFF3 format) were downloaded from NCBI (genome accession number AE006468.2 and plasmid accession number AE006471.2). *Samtools* were used to merge the bam files (Li et al., 2009). *Tophat2* was used to map the trimmed paired-end and single-end reads to the *Salmonella* genome (Kim et al., 2013). Moreover, small RNA sequences from (Srikumar et al., 2015) were mapped to the LT2 genome. Only sequences that had 100% sequence identity over the full length on LT2 strain were retained. Expression analysis by counting reads per genomic feature from the *Salmonella* annotation file was performed using the *featureCounts* tool from the *Subread* package (Liao et al., 2014). Read counts per genomic feature were subsequently subjected to statistical analysis with R. All libraries were depleted of rRNA *in silico* to allow intracellular/extracellular comparisons, except if stated otherwise.

#### Count Normalization, PCA and ANOVA

Normalization among samples and conditions was performed using the *DESeq2* package (Love et al., 2014), i.e., results were normalized for library sizes but not for gene length as the purpose of our analysis was to compare the expression of identical genes in different conditions. Moreover, rRNA was removed *in silico* from all datasets (otherwise stated) to avoid potential biases in the comparisons between intracellular and OMV-associated RNAs as they were experimentally removed from intracellular fractions only. Principal component analyses (PCA) were obtained with the *plotPCA* function from *DESeq2*. Analysis of variance (ANOVA) was performed with subsequent *post hoc* analysis to test if significant differences in relative amounts of exported RNA classes between the different culture conditions exist, using Tukey’s range test from the *multcomp* package (Hothorn et al., 2008) with FDR-adjusted *p*-values (Benjamini–Hochberg procedure). To produce the scatterplots in **Figure 7**, sum normalization was performed using the *decostand* function from the *vegan* package (Oksanen et al., 2018).

#### Determination of Exported and Enriched RNAs

Exported RNAs associated to OMVs were filtered for a normalized count over 1, averaged from biological triplicates. Enriched RNAs associated to OMVs were determined using *DESeq2* (Love et al., 2014), calculating the differential presence between intracellular and OMV-associated fractions for each condition. Transcripts with an average normalized number of extracellular reads over 5 for a given set of triplicates, a log_2_ Fold Change_(OMV/intracellular)_ over 2, and a corresponding FDR-adjusted *p*-value under 0.05 were considered as enriched.

#### Read Coverage Analysis

RNA coverage from sequencing data was visualized with the *Integrative Genomics Viewer* software (v2.4.8) using default parameters (Robinson et al., 2011).

#### Figure Generation

**FIGURE 1:**
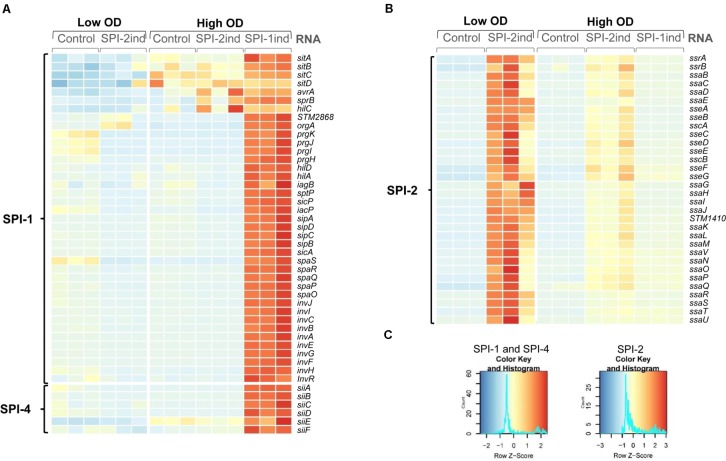
Activation of SPI-1 and SPI-2 by different *in vitro* culture conditions. Heatmaps showing relative expression of SPI-encoded genes in different conditions and biological replicates used in the study. RNA-Seq data of the intracellular fractions were normalized using *DESeq2*. Red or blue colors indicate a higher or lower expression, respectively. **(A)** Bacteria grown in SPI-1ind medium (LB) until stationary phase (OD_600nm_ = 1.6) express SPI-1 and SPI-4 genes whereas growth in SPI-2ind medium or in control condition (PCN) represses these Pathogenicity Islands as expected. **(B)** For SPI-2 genes, the *in vitro* conditions with low pH and limited phosphate availability clearly trigger activation of the island in low OD, and to a lower extent in high OD. **(C)** Color keys and histograms for the heatmaps shown in **(A,B)**.

**Figure 1** was generated using the *heatmap.2* function from the *gplots* package, scaled by row. **Figure 8** was generated using the *UpSet R* package (Conway et al., 2017). Other graphs were obtained with *ggplot2* (Wickham, 2009).

### PCR Validation

To confirm results obtained by RNA sequencing and to show that RNAs of interest are present in or on OMVs in full-length, PCR validation was performed for distinct RNAs (**Supplementary Dataset C**) on independent replicates from high OD conditions.

#### cDNA Library Synthesis

New *S*. Typhimurium intracellular samples and purified OMV fractions were obtained from SPI-1ind, SPI-2ind, and Control conditions at high OD, with two biological replicates per condition. Intracellular and OMV-associated RNA was obtained as previously described with the following differences: the initial culture volume was 0.75 l per replicate instead of 1.5 l; RNA extractions were performed using TRIzol Reagent (Invitrogen); cDNA libraries were prepared from 1 μl of OMV-associated RNA or 100 ng of intracellular RNA with the SuperScript III First-Strand Synthesis System (Invitrogen) using random hexamer primers according to the manufacturer instructions and without further cDNA fragmentation step.

#### Full Length RT-PCR and Visualization of the Amplicons

Polymerase Chain Reaction was performed using Phusion Green Hot Start II High-Fidelity PCR Master Mix (Thermo Scientific). A set of primers was designed to amplify the totality of the targeted Open Reading Frame (from start to stop codon), or the totality of the selected ncRNAs (list of targets and primers available in **Supplementary Dataset C**). 0.5 μl of cDNA library was used for each 20 μl PCR reaction as template, together with 1 μM of primers. Intracellular libraries were used as positive control, and cDNA synthesis reactions were performed on OMV-associated RNA without adding reverse transcriptase (RT) as a negative control to check for the presence of residual genomic DNA. Three different Touch-Down PCR programs were used, depending on the target length and differed only by their extension time (15 s per cycle for small targets under 500 bp, 45 s for medium targets between 500 and 1500 bp, and 70 s for large targets above 1500 bp). The 3 first cycles used an annealing temperature of 72°C during 7 s, which was lowered by 3°C steps every 3 cycles until reaching 57°C for the last 20 cycles. This approach was used to ensure a good selectivity without the need of further optimization. The size of PCR products was checked by running 10 μl of PCR reactions on an agarose gel prepared with 3% w/v TopVision Agarose (Thermo Scientific) in Tris-Borate-EDTA buffer (89 mM Tris, 89 mM Boric acid, 2 mM EDTA, pH = 8) for small size products or on a 1% w/v agarose gel in Tris-Acetate-EDTA buffer (40mM Tris, 20 mM acetic acid, 1 mM EDTA, pH = 8) for large size products, using the MassRuler DNA Ladder Mix (Thermo Scientific) as a size guide. In the case of 3% gels, the agarose solution was autoclaved to ensure an optimal dissolution before pouring the gel. Electrophoresis was run at 130 V during 200 min (3% gels) or 100 V during 100 min (1% gel) and DNA was stained with ethidium bromide. Pictures were acquired with a Canon EOS 1200D camera coupled to a BioDocAnalyzer (Biometra).

#### q-RT-PCR

In addition to the PCR, qPCR was used to confirm for some RNAs their higher signal in RT-positives (+RT) libraries compared to RT-negative (-RT) controls, using absolute comparison between *C*_t_ values from both - and + RT conditions. As cDNA synthesis was performed with an identical protocol and the same quantity of template and as all qPCR runs had been performed on the same plate within the same instrument, we assumed that a greater *C*_t_ for a given gene in the +RT library compared to the -RT control is indicative for the presence of the targeted RNA. qPCR reactions were performed on technical duplicates for each biological replicate, using PerfeCTa SYBR^®^ Green SuperMix (QuantaBio). The primers used are listed in **Supplementary Dataset C**. 0.75 μl of cDNA was used as a template for each 10 μl reaction, which was carried out with 100 nM of primers. Cycling conditions were as follow: initial denaturation 5 min at 95°C, 45 cycles of 15 s at 95°C, 30 s at the adapted annealing temperature (see **Supplementary Dataset C**), 30 s at 72°C, and a melting curve consisting of 5 s at 95°C and 1 min at 55°C. A first gradient run was performed on intracellular cDNA synthesis to select the optimal annealing temperature and to ensure that no coproducts were formed by analyzing the melting curves. The data was treated and analyzed with the LightCycler480 software to determine the *C*_t_. Results with *C*_t_ equal or above 40 were considered as negative. Only technical duplicates with less than 5% variation were considered as reliable. For two given technical duplicates, if the difference between the average of +RT *C*_t_ and the average of -RT *C*_t_ is equal or superior to 3, the difference is considered to be significant (eight times more cDNA than residual gDNA in the sample).

#### Proteinase and RNase Protection Assay

To analyze if some nucleoprotein aggregates were co-purified with our OMV preparations, we performed a digestion protection assay (Shelke et al., 2014). OMV isolation was performed from the SPI-1ind condition as described in Section “OMV Purification and RNA Extraction,” except that a supplemental enzymatic step was added before density gradient ultracentrifugation. The crude OMV preparation was divided into four fractions. One was kept untouched (Untreated). The second was subjected to the same protocol as well as the third and fourth fraction without any enzyme added (Control). The third was digested by RNaseA (QIAGEN, 7000 U/ml) at 0.1 μg.μl^-1^ and 37°C during 20 min. The fourth was digested by ProteinaseK (Thermo Scientific, >600 U/ml) at 0.05 μg.μl^-1^ and 37°C during 30 min, followed by proteinase inactivation (adding 5 mM phenylmethylsulfonyl, Sigma-Aldrich) and RNAseA digestion. The protocol was then followed until the cDNA libraries from the OMV-associated RNAs were obtained. End-point PCR was then performed with the same primers and conditions used previously for *SsrS*, *CsrC*, *10Sa*, and *rnpB* genes. Resulting samples were deposited on a 3% w/v agarose gel in TBE buffer, subjected to electrophoresis and stained with Ethidium Bromide. The efficiency of RNase digestion was confirmed using an intracellular RNA extract (10 ng) subjected to the same protocol (**Supplementary Figures S5B,C**). The efficiency of proteinase digestion was confirmed on an intracellular protein extract (5 μg), which had been digested under the same conditions (**Supplementary Figure S5D**).

## Results

Outer membrane vesicles originating from *S.* Typhimurium cultured in five distinct conditions, with three biological replicates per condition, were purified and the associated RNAs were sequenced, together with their respective intracellular counterparts.

### RNA Is Associated With *S.* Typhimurium OMVs

RNA sequencing (RNA-Seq) was performed on the RNA isolated from OMVs secreted by *S.* Typhimurium in order to study their RNA composition. A significant number of transcripts was found in OMVs under all conditions (control Low and High OD, SPI-2ind Low and High OD, SPI-1ind High OD). Reads were mapped to the *S.* Typhimurium LT2 genome and pSLT plasmid and annotated using NCBI, and sRNA supplemental annotations from (Srikumar et al., 2015). Basic statistics of the RNA-Seq data are displayed in **Supplementary Dataset A** for low and high OD samples. Briefly, intracellular samples had roughly all around 10 million reads mapped to the target genome. For OMV samples, the number of reads and mapping percentages were lower, especially for SPI-1ind samples. The main contaminating RNAs mapped to *Agrobacterium*, *Rhizobium*, and *Achromobacter* taxa, which are common contaminants originating from column purification kits, when dealing with low abundance samples (Susilawati et al., 2016; Heintz-Buschart et al., 2018). These contaminating RNAs were thus added during the isolation protocol of RNAs and were originally not present in the *S.* Typhimurium cultures. As they did not originate from *Salmonella*, they were not taken into account for any further analysis. However, all OMV libraries were composed of reads mapping to most of the annotated genes in the *Salmonella* LT2 genome, leading to a mean of 3601 ± 545 expressed genes with an average count over 5 per condition (see **Supplementary Figure S6A**). As our annotated genome comprised 4964 genes, it means that on average, around 73% of the genes were expressed and identified in/on OMVs, even if it does not imply that these were native transcripts.

### SPI-1 and SPI-2 Are Expressed in Their Respective *in vitro* Culture Condition

To confirm that the specific *in vitro* culture conditions chosen to mimic different life cycle steps of *S.* Typhimurium during host–pathogen interaction effectively induced the expression of the desired *Salmonella* pathogenicity island (SPI), we looked at the intracellular expression of the different SPI genes. As expected, SPI-1 expression was increased in SPI-1ind high OD conditions (**Figures 1A,C**), in agreement with previous findings (Kröger et al., 2013). The activation of SPI-4, which is controlled by SPI-1, was also observed. Concerning SPI-2, the expression of the pathogenicity island was favored in SPI-2ind conditions especially at low OD (**Figures 1B,C**). Residual expression was also present in stationary phase for the same medium (**Figures 1B,C**).

### *S.* Typhimurium OMVs Isolated From the Different Culture Conditions Display a Distinct RNA Profile

**FIGURE 2:**
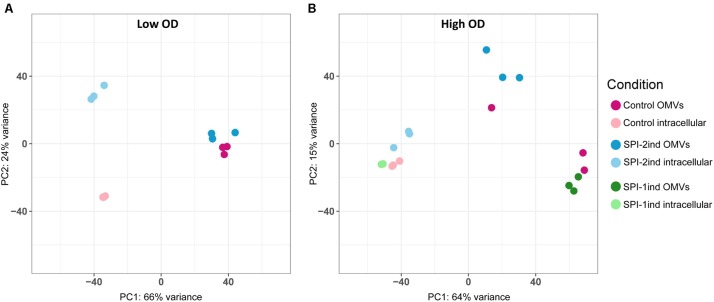
Principal component analysis. **(A)** Low OD samples. **(B)** High OD samples. Both intracellular and OMV-associated fractions are displayed. Normalized counts obtained with the *DESeq2* package were used as input, omitting ribosomal RNAs, which were experimentally depleted for intracellular fractions, and removed *in silico* for OMV fractions. OMV and intracellular fractions are displayed in dark and light colors, respectively.

In order to acquire a broad view of the RNA expression profiles in the different samples, we performed Principal Component Analyses (PCA) on low and high OD samples (**Figures 2A,B**). The results confirm distinct clustering of the intracellular samples and, to a lesser extent, the OMVs triplicates. However, a general impression emerges from this data: most of the variance in these profiles is between the intracellular and OMV samples, which clearly points to a selective packaging of the exported RNAs. A difference is also observed in the high OD samples between SPI-1ind OMVs versus SPI-2ind OMVs triplicates. It is notable that these are conditions that mimic different infection steps (**Figure 2B**). Moreover, when only RNAs (rRNA included) associated with OMVs within SPI-1ind and SPI-2ind conditions were used for a PCA, we could observe that 79% of the variance was explained by the growth conditions (**Supplementary Figure S7A**). When only intracellular RNAs from these same conditions were compared, 81% of the variance was explained (**Supplementary Figure S7B**). Thus, the OMV-RNA complement reflects the adaptation of *S.* Typhimurium to its environment.

### The Proportion of RNA Classes Associated With the OMVs Varies According to the Environmental Condition

**FIGURE 3:**
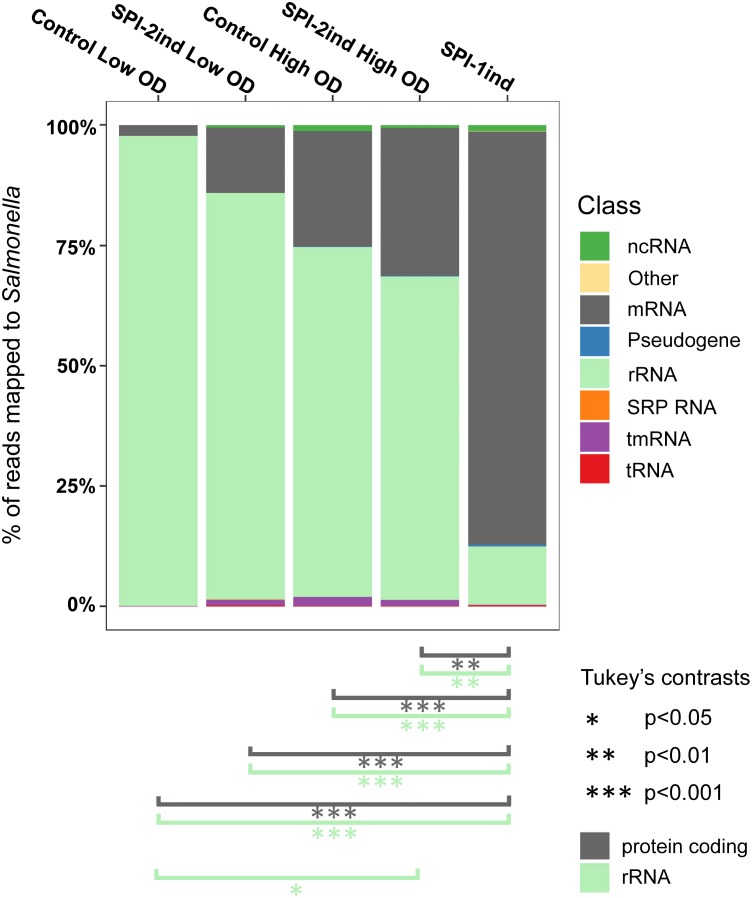
Relative composition of the OMVs RNA cargo for each culture condition. Individual colored bars represent the relative amount of each RNA class in the respective OMV RNA-Seq data (average of the biological triplicates). Number of reads have been normalized with *DESeq2*. RNA classes are defined from the *Salmonella* LT2 genome annotation (NCBI accession number AE006468.2) or pSLT plasmid annotation (NCBI accession number AE006471.2), with supplemental small RNA annotations from Srikumar et al. (2015). The existence of significant proportion differences among conditions for a specific RNA class was assessed by one-way ANOVA and *F*-test. Significant differences for rRNA and mRNA classes between conditions are indicated under the graph, determined by Tukey’s range test. Further details for other RNA classes can be found in **Supplementary Dataset B**.

When analyzing the RNA classes present in OMV-associated fractions, it became clear that every class of transcript is exported, including rRNAs, mRNAs, tRNAs and others ncRNAs (**Figure 3** and **Supplementary Dataset B**). One can observe that for all but the OMVs originating from SPI-1ind, the vast majority of exported RNAs were rRNAs, as it has already been observed in other species [e.g., in *Escherichia coli*, see (Blenkiron et al., 2016)]. However, in the case of SPI-1ind grown bacteria, the proportion of rRNA associated to OMVs was much lower, down to only 12.1 ± 15.2%. Concomitantly, we observed an increase in the mRNA relative amount up to 85.9 ± 15.1%. Despite the variance observed between triplicates, both of these values were significantly different from their respective counterpart in other conditions as determined by Tukey’s range test (**Figure 3**). Thus, it seems that the balance between rRNAs and mRNAs depends on the environmental conditions (pseudogenes followed the same trend than mRNAs). This result needs to be put in perspective, taking into account the lower sequencing depth obtained for the SPI-1ind OMV samples (**Supplementary Dataset A**). On the contrary, no significant differences were detected concerning tmRNA, tRNAs, and others ncRNAs, which appear to have been exported the same way in all conditions (see all ANOVA and Tukey’s test results in **Supplementary Dataset B**). Concerning tRNAs, we found a limited relative amount of tRNAs (<0.2% for all conditions). These were probably exported, but mainly found in the small RNA fraction (<50 bp) and therefore would have probably been more visible in a small RNA-Seq library, which was not included within the present study (Ghosal et al., 2015; Blenkiron et al., 2016).

### Native and Processed and/or Degraded RNA Co-exist Among OMV-Associated RNAs

Most of the existing studies on OMV-associated RNAs have emphasized non-coding small and micro RNAs in cell-to-cell information transfer (Gong et al., 2011; Dauros-Singorenko et al., 2018; González Plaza, 2018). Concerning longer transcripts, both their biological pertinence and integrity must be questioned. As we found a good proportion of reads mapping to larger RNAs in our samples, we investigated the apparent state of degradation of some OMV-associated RNAs.

#### Sequencing Coverage Analysis

**FIGURE 4:**
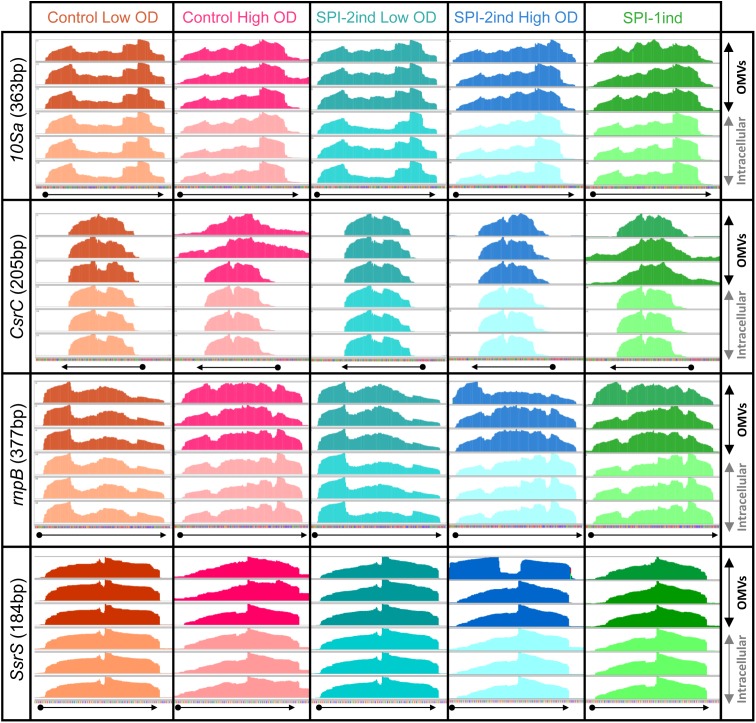
Visualization of intracellular and OMV-related read coverage plots of distinct sRNAs in distinct culture conditions. RNA coverage from sequencing data was visualized with the Integrative Genomics Viewer software (v2.4.8) using default parameters (Robinson et al., 2011). Each plot represents the raw number of reads mapped along the observed sequence, automatically scaled relatively to the highest number of read existing in this portion of the genome. The three plots in dark colors at the top of each window represents the sequencing coverage of OMV-associated fractions, in biological triplicate for each condition. On the contrary, the three plots in lighter colors at the bottom of each window show the coverage for the same RNA but from the corresponding intracellular fractions. The arrows under each window precise the genes positions and orientations, according to the data extracted from the *Salmonella* LT2 genome annotation (NCBI accession number AE006468.2) or pSLT plasmid annotation (NCBI accession number AE006471.2). The identical coverage patterns observed in both fractions for the displayed genes stand for a native export through OMVs without specific processing or degradation.

To obtain a first insight into the apparent state of degradation of exported transcripts, we investigated the sequencing coverage of various genes in the intracellular and OMV-associated RNA-Seq data. Results show a variety of coverage profiles, falling into three broad categories. The first group consisted of transcripts showing identical patterns between intracellular and OMV-associated RNAs, and included *10Sa*, *CsrB*, *CsrC*, *rnpB*, and *SsrS* (visible in **Figure 4** and **Supplementary Figure S8A**). For these RNAs, the identity was almost constant through all media conditions tested. The 16S and 23S rRNA also felt into that group with few changes between intracellular and OMV-specific coverages (intracellular rRNA remaining after experimental rRNA depletion was sufficient to obtain a good coverage, see the example of *rrlA* in **Supplementary Figure S8B**). The second group of transcripts was fully covered by sequencing, but with sometimes very different read densities between the two fractions. For instance, it comprised *ffs* (except for SPI-2ind LowOD where patterns were identical), *gogB* in High OD, *IsrA* in SPI-1ind and Control High OD conditions, *IsrB-1/2*, *nanH*, *pagC*, *pipA*, *sipADCB* in SPI-1ind and Control HighOD, *slrP*, *pSLT035*, *sseC*, *sseI*, *tae4* (*STM0277*), *STM0289*, *STM0972* (*sopD2*). Some examples are displayed in **Supplementary Figures S8B,C**. These differences between intracellular and extracellular fractions might be explained by the low abundance of some RNA in the extracellular fraction, but drastic changes could also come from preferential export of processed RNAs or degradation products. The third category consisted of RNAs poorly or not detected in OMVs, meaning they were heavily degraded or their export was almost inexistent.

#### OMV-Associated RNAs Are Either Full Length, Processed and/or Degraded

**FIGURE 5:**
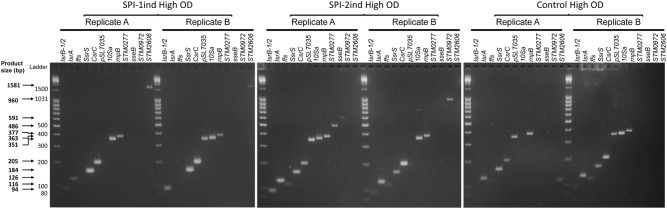
Integrity analysis of selected High OD exported RNAs by full-length RT-PCR. After OMVs isolation and RNA extraction as described in Section “Materials and Methods,” cDNA synthesis was performed. End-point PCR was then conducted with primers flanking the coding sequence (mRNA) or the full sequence (ncRNA) of 30 selected RNAs. Resulting samples were deposited on a 3% w/v agarose gel in TBE buffer, subjected to electrophoresis and stained with Ethidium Bromide. Only transcripts that were amplified as full-length amplicons are shown (12 out of 30 selected RNAs that were tested). Two biological replicates for SPI-1ind, SPI-2ind High OD and Control High OD conditions are visualized. Primers can be seen in **Supplementary Dataset C**, –RT controls are displayed in **Supplementary Figure S9** and a positive control of full-length amplifications performed on intracellular RNAs is visible in **Supplementary Figure S10**.

As some exported RNAs appeared to be complete transcripts according to their coverage profiles, we designed a set of primers to validate their integrity experimentally by PCR amplification. The full coding-sequence of 20 mRNAs and the totality of 10 ncRNAs sequences were targeted and amplifications were performed on high OD condition samples (**Supplementary Dataset C**). 12 RNAs were found to be amplified in their full-length from our cDNA libraries in at least one condition: 7 ncRNAs (*IsrB-1/2, ffs, IsrA, SsrS, CsrC, 10Sa, rnpB*) and 5 mRNAs [*pSLT035*, *STM0277* also called *tae4*, *sseB*, *STM0972* (*sopD2*), *STM2606*] (**Figure 5**).

Full-length mRNAs were amplified in only one out of the two replicates analyzed (for instance, *sseB* and *STM0972* in the case of SPI-2ind High OD). *STM0277* was also detected in SPI-2ind High OD but not in SPI-1ind, whereas a greater number of reads was detected in OMVs from the later by sequencing. Interestingly, we observed a tendency toward the amplification of the smaller sized RNAs compared to the larger ones (**Figure 5** and **Supplementary Dataset C**). This trend might be due to a higher degradation predisposition of larger RNAs compared to small RNAs, which are usually more structured and therefore more stable. Especially, protein-associated ncRNAs such as *ffs* (SRP RNA binding to Ffh and routing nascent proteins to the membrane), *rnpB* (RNA catalytic component of the RNaseP ribozyme involved in pre-tRNAs maturation), *SsrS* (6S RNA that binds and regulates the σ_70_ transcription factor) or *10Sa* (tmRNA associated to SmpB) were remarkably conserved in OMVs, in complete agreement with the RNA sequencing results (**Figures 4**, **5** and **Supplementary Figure S8A**). Fragments originating from tmRNA, SRP-RNA or 6S RNA were already reported to be present in the extracellular complement of *E. coli*, but completeness was not assessed (Ghosal et al., 2015).

**FIGURE 6:**
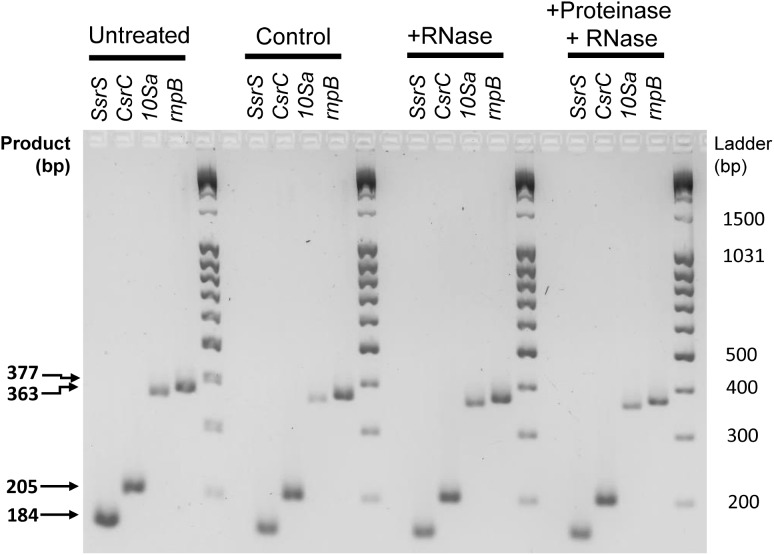
RNase protection assay of selected protein-associated sRNAs isolated from OMVs. OMV isolation and enzymatic digestions were performed from SPI-1ind condition as described in Section “Materials and Methods (see section “OMV Purification and RNA Extraction”).” Crude OMV preparation was divided in four fractions. One was kept untouched (Untreated). The second was subjected to the same protocol than the third and fourth fraction without any enzyme added (Control). The third was digested by RNaseA. The fourth was digested by ProteinaseK followed by proteinase inactivation and RNAseA digestion. The protocol was then followed as described in Section “OMV Purification and RNA Extraction” until cDNA libraries from OMV-associated RNAs were obtained End-point PCR was then conducted with the same primers and conditions used previously, for *SsrS*, *CsrC*, *10Sa*, and *rnpB* genes. Resulting samples were deposited on a 3% w/v agarose gel in TBE buffer, subjected to electrophoresis and stained with Ethidium Bromide. Most of the signal is still present after enzymatic digestion, showing that the RNAs are protected by the vesicle membrane. –RT controls and positive controls for enzymatic digestions can be seen in **Supplementary Figure S5**.

Hence, one may consider that the presence of these RNAs in our samples might be due to a “contamination” with nucleoprotein complexes occurring during the purification process of the vesicles. This issue has already been identified in the Extracellular Vesicle field, resulting in artificial enrichment of the OMV-associated cargo with protein-associated RNAs (Mateescu et al., 2017). Therefore, we performed an RNase and proteinase protection assay on OMVs isolated from SPI-1ind condition (**Figure 6**; -RT controls are represented in **Supplementary Figure S5A**). As neither RNase nor proteinase followed by RNase treatment prevented RT-PCR on *SsrS*, *CsrC*, *10Sa*, and *rnpB*, we assume that these ncRNAs were packaged inside the OMVs instead of being passively enriched during vesicles purification. However, this association might also occur when these are bound to their partner proteins explaining their stability. Note that in the same digestion conditions, intracellular RNA or protein extract was almost completely digested and only *rnpB* gave a low signal by RT-PCR after RNase treatment (**Supplementary Figures S5B,C**).

Some -RT controls showed a remaining DNA contamination (**Supplementary Figure S9**) despite the DNAseI digestion step before cDNA synthesis. Therefore, we performed qPCR on +RT and -RT samples for several genes and confirmed that the amount of cDNA was greater in +RT than in -RT for *ffs*, *CsrC*, *rnpB*, *sseB*, and *STM0972* (*sopD2*) (**Table 1**). For *sseB* and *STM0972*, both replicates were positive contrary to the full-length PCR, indicating that the transcripts were present but not in their native form or the end-point PCR was not sensitive enough to detect them on a gel.

**Table 1:** q-RT-PCR *C*_t_ comparison for five selected genes between cDNA libraries generated from OMV-extracted RNA isolated from SPI-1ind, SPI-2ind High OD and Control High OD conditions.

	*ffs*		*CsrC*		*rnpB*		*sseB*		*STM0972*	
					
	*C*_t_ +RT	*C*_t_ -RT	*C*_t_ +RT	*C*_t_ -RT	*C*_t_ +RT	*C*_t_ -RT	*C*_t_ +RT	*C*_t_ -RT	*C*_t_ +RT	*C*_t_ -RT
SPI-1ind A	**33,32**	n.s.	**30,47**	37,75	**30,58**	n.s.	–	–	–	–
	**34,84**	n.s.	**30,8**	n.s.	**30,6**	n.s.	–	–	–	–
SPI-1ind B	**29,77**	n.s.	**25,97**	40	**26,07**	36,68	–	–	–	–
	**30,14**	n.s.	**25,93**	n.s.	**26,09**	40	–	–	–	–
SPI-2ind A	**37,02**	n.s.	**29,44**	n.s.	**33,5**	n.s.	**30,28**	40	**34,46**	n.s.
	**36,04**	n.s.	**29,56**	n.s.	**33,1**	n.s.	**30,2**	38,24	**33,75**	n.s.
SPI-2ind B	**36,81**	n.s.	**30,02**	n.s.	**33,27**	37,94	**34,7**	n.s.	**34,89**	n.s.
	**37,69**	n.s.	**29,79**	n.s.	**33,65**	37,82	**33,7**	n.s.	**35,84**	n.s.
Control A	**36,42**	n.s.	**34,37**	n.s.	**35,2**	n.s.	–	–	–	–
	**36,6**	n.s.	**33,57**	n.s.	**36,45**	n.s.	–	–	–	–
Control B	**37,11**	n.s.	**34,06**	n.s.	**34,3**	n.s.	–	–	–	–
	**38,53**	n.s.	**33,15**	n.s.	**34,29**	n.s.	–	–	–	–


*Libraries were constructed with (+RT) and without (-RT) adding the reverse transcriptase to confirm that DNA contamination was not responsible for bands amplified by full-length PCR. qPCR was performed on biological replicates for each condition, with technical duplicates. Results with C_*t*_ equal or above 40 were considered as negative. Only technical duplicates with less than 5% variation were considered as reliable. For two given technical duplicates, if the difference between the average of +RT C_*t*_ and the average of -RT C_*t*_ is equal or superior to 3, the difference is considered as significant (eight times more cDNA than residual gDNA in the sample). n.s., no signal after 45 cycles, meaning no amplification was detected. Water controls always led to n.s. Primers information is displayed in **Supplementary Dataset C**.*

In contrast to the genes highlighted above, no full-length transcripts associated to OMVs could be detected for the following genes: *STnc440, STM0278, STM1026, pagC, spvD, STM0292, ydgH, sseI, AmgR, sipC, nanH, gogB, sseC, sipB, sipA, STM0289, slrP*, and *STM0291* (**Supplementary Dataset C**).

### Some RNAs Are Over-Represented in/on OMVs Compared to Their Intracellular Counterparts

**FIGURE 7:**
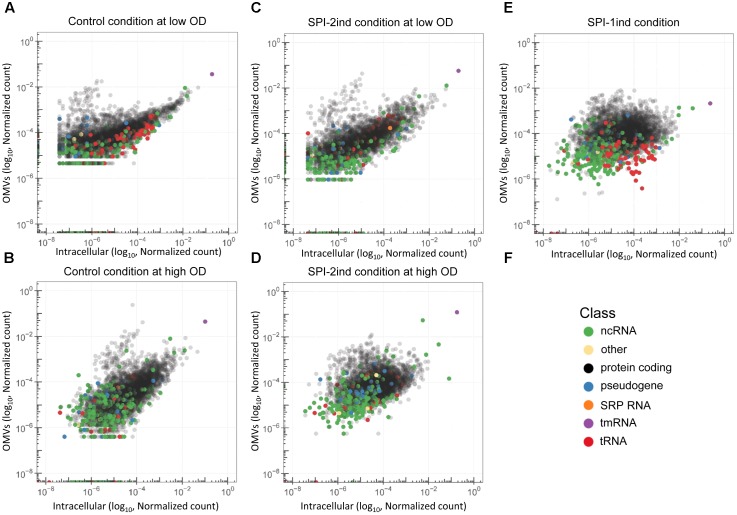
Differential abundance of RNAs in OMVs and their intracellular counterparts. **(A–E)** Scatterplots showing the reads repartition within a given growth condition. Each dot represents the relative abundance of an expressed gene in or attached to the OMVs (vertical axis) and in the intracellular (horizontal axis) fraction for each growth condition used in this study. RNA annotation from the *Salmonella* LT2 genome (NCBI accession number AE006468.2) or pSLT plasmid (NCBI accession number AE006471.2) is precized by a color code. Number of reads were normalized by sum normalization for each condition using the decostand function from the vegan R package. Ribosomal RNAs, which were experimentally depleted for intracellular fractions, and removed *in silico* for OMV fractions, are omitted. **(F)** Color legend used to underline the RNA class of each annotated transcript in the graphs.

We could show that different culture conditions induce different OMV-associated RNA export. In order to study the RNA cargo present in the vesicles in detail, we performed pairwise comparisons between intracellular and OMV fractions among our five culture conditions. Simple scatterplots of normalized counts already provided a good view of the existence of transcripts that were enriched in/on OMVs compared to the intracellular fraction (**Figure 7**). In this context, it is important to note that we did not have the possibility to distinguish between full-length or processed RNAs which remain functional and degradation products for each individual transcript enriched in/on OMVs as described and explained in section “Native and Processed and/or Degraded RNA Co-exist Among OMV-Associated RNAs.” As, however, this remained true for all the different culture conditions, we performed pairwise comparisons between intracellular and OMV-associated RNAs among our five culture conditions, in order to obtain a global overview of RNA sequences which were differentially expressed among the different culture media. The RNAs that were enriched in/on OMVs compared to their intracellular counterpart were mainly coding sequences and to a lesser extent, ncRNAs (**Figure 7**, in black and green, respectively). On the contrary, tRNAs seemed more internalized for the studied transcript libraries (**Figure 7**, red). Note that rRNAs are not displayed here as the intracellular samples had been experimentally depleted of the latter and thus intracellular versus OMV RNA sample comparisons became impossible. The pattern was clearly dependent on the condition, with a wider spread in high OD conditions and especially in SPI-1ind (**Figures 7C–F**). Interestingly, the translation rescuing RNA tmRNA (*10Sa*), which was experimentally proven to be packaged as full-length transcript (**Figures 5**, **6**) was always highly represented in both fractions. As *10Sa* binds other proteins for its activity, and among them elongation factors or ribosomal proteins, its OMV association could be favored by the presence of the elongation factor EF-G in the vesicles (Bai et al., 2014; Himeno et al., 2014). However, no trace of its main protein partner SmpB has been reported to date.

**FIGURE 8:**
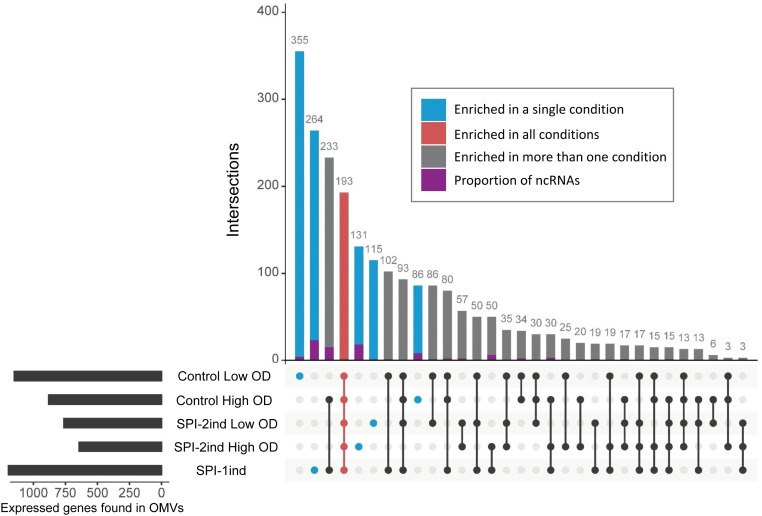
Repartition of enriched RNAs in/on OMVs isolated from the different analyzed *in vitro* culture conditions. UpSet plot representing the number of enriched RNAs shared between different conditions. One unit represents one expressed gene. The graph was generated using the UpSet R package. Enriched RNAs were determined using *DESeq2*, calculating differential presence between intracellular and OMV-associated fractions for each condition. Expressed genes with an average number of reads over 5 for a given set of triplicates, a log_2_ Fold Change over 2, and a corresponding adjusted *p*-value under 0.05 were selected. ncRNA class attribution is defined from the *Salmonella* LT2 genome annotation (NCBI accession number AE006468.2) or pSLT plasmid annotation (NCBI accession number AE006471.2). Moreover, small RNA annotations from Srikumar et al. (2015) were mapped to the LT2 genome. Only sequences that had 100% sequence identity over the full length on LT2 strain were kept. Identical datasets were used for the generation of **Figure 8** and **Supplementary Figure S6B**.

In order to define more precisely the OMV-enriched transcripts in comparison to their intracellular counterpart, a differential expression analysis with the *DESeq2* package (Love et al., 2014) was performed. We chose to select RNAs with a log_2_ Fold Change (logFC) (OMV/intracellular) ≥ 2, a *p*-value < 0.05 (Welch’s *t*-test), and an averaged number of reads per condition ≥ 5 in order to ensure a stringent selection of “enriched RNAs.” Henceforth, all RNAs that fulfill the aforementioned criteria are referred to as enriched RNAs, whereas we refer to exported RNAs when a given RNA is associated with OMVs, but not necessarily enriched compared to its intracellular counterpart. In each media condition, a significant number of transcripts was indeed selectively exported (**Figure 8**). In total, we found 1151 enriched transcripts in Control Low OD condition, 763 in SPI-2ind Low OD conditions, 881 in Control High OD condition, 643 in SPI-2ind High OD condition and 1196 in SPI-1ind condition (detailed lists are available in **Supplementary Dataset D**). A smaller core set of 193 RNAs was enriched in all conditions tested, with the notable absence of ncRNAs in this set, these being mainly found in condition-specific groups. However, these group sizes were very low compared to the totality of the RNA cargo, which originated from 4670 genes in total, from which 2217 were found in all the conditions tested (**Supplementary Figure S6A**). This difference may underline the dual nature of OMV-RNA association, reflecting the intracellular state of the transcriptome through global packaging, but also containing specific sets of read fragments mapping to distinct RNAs that depend or not on the current environmental state.

#### Virulence Effectors Related RNAs Are Among the Selected Cargo for Export

**FIGURE 9:**
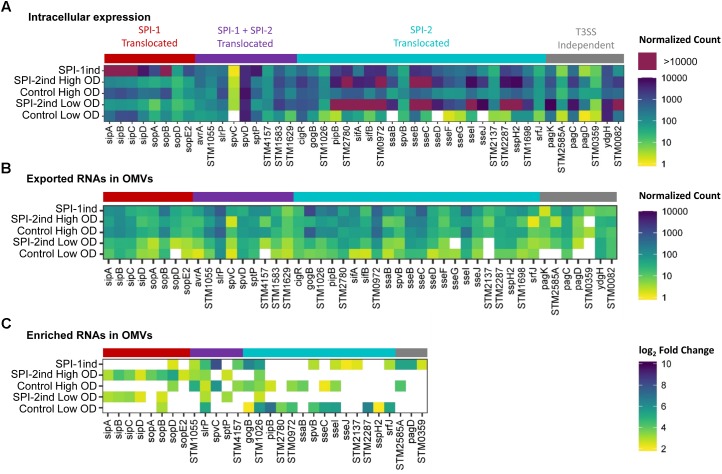
Intracellular expression, export and enrichment of OMV-associated effectors mRNAs. List of translocated effectors involved in virulence and infection of the host by *Salmonella*, regrouped by their known export route (Niemann et al., 2011; Kidwai et al., 2013; LaRock et al., 2015). T3SS, Type 3 Secretion System. **(A,B)** Mean count among triplicates in intracellular **(A)** or in OMV fractions **(B)** is displayed as a colored square, from low (yellow) to high count (dark blue). Counts above the upper limit of the scale are colored in purple. Counts were normalized using *DESeq2* (Love et al., 2014), and filtered for a minimum averaged count of one read per biological triplicate. **(C)** Enriched RNAs associated to OMVs were determined using *DESeq2*, calculating differential presence between intracellular and OMV-associated fractions for each condition. Expressed genes with an average number of reads over 5 for a given set of triplicates, a log_2_ Fold Change over 2, and a corresponding adjusted *p*-value under 0.05 were selected. The log_2_ Fold Change is displayed as colored squares from 2 (yellow) to 10 (dark blue).

As we validated the association of several full-length virulence-related mRNAs with OMVs (**Figure 5**, **Table 1** and **Supplementary Dataset C**) and as most of the effectors were covered over the whole length of their gene (**Supplementary Figures S11A–E**), we looked at the more than 40 effectors known to be secreted by *Salmonella* during invasion and infection (Niemann et al., 2011; Yoon et al., 2011a; Kidwai et al., 2013; LaRock et al., 2015). Intracellular expression, OMV- exported and enriched effectors are displayed in **Figure 9**. As expected, the intracellular expression of secreted molecules was linked to their known export route: SPI-1 translocated effectors were more expressed in non-SPI-2ind conditions and vice-versa (**Figure 9A**). However, even if almost all effectors’ mRNAs were found associated with OMVs in every condition, the OMV export route appeared to be more important when the corresponding T3SS (Type 3 Secretion System) was absent (**Figure 9B**). This was confirmed by the analysis of enriched effectors RNAs, where a distinct pattern did exist (**Figure 9C** and **Supplementary Dataset E**): SPI-1 translocated effectors seemed enriched in/on vesicles produced in SPI-2ind conditions (i.e., *sip* genes), whereas SPI-2 translocated ones were enriched in/on OMVs originating from non-SPI-2ind conditions, i.e., Control and SPI-1ind (i.e., some *sse* transcripts). Interestingly, we also identified prophage-encoded effectors. For instance, GogB is a secreted effector originating from Gifsy-1 prophage that can be translocated through both the SPI-1 and SPI-2 T3SS, but which is mainly expressed under SPI-2 control by *ssrB* (Coombes et al., 2005). It has an anti-inflammatory effect by interacting with ubiquitin ligase in the host, limiting tissue damage during infection (Pilar et al., 2012). It was also associated with OMVs in all conditions and singularly enriched in the controls and mainly SPI-1ind (logFC = 6.39, *p* < 0.001), emphasizing its multiple roles in pathogenicity as this protein might be involved in regulating immune processes from an early stage of the host–pathogen interaction. One can also note the stable presence of transcripts of T3SS-independent effectors such as *ydgH* (or *sssB*) and *STM0082* (or *srfN*) (Eletsky et al., 2014). No enrichment of T3SS-independent effectors was observed in OMVs isolated from SPI-2ind conditions even though their intracellular expression is increased in acidic medium and they are suspected to be exported through OMVs to the host cell (**Figure 9B**, Yoon et al., 2011b).

**FIGURE 10:**
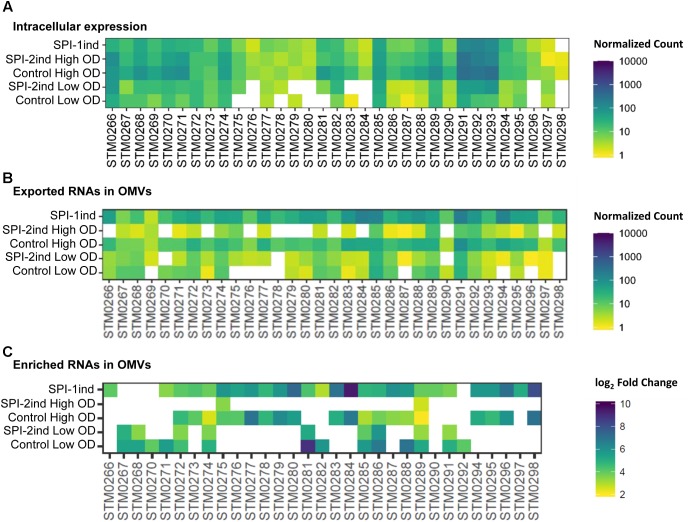
Intracellular expression, export and enrichment of SPI-6 encoded mRNAs. For each enriched SPI-6 mRNA, the intracellular expression, the presence and the enrichment in OMVs (if applicable) are displayed. **(A,B)** Mean count among triplicates in intracellular **(A)** or OMV fractions **(B)** is displayed as a colored square, from low (yellow) to high count (dark blue). The counts were normalized using *DESeq2* and filtered for a minimum averaged count of one read per biological triplicate. **(C)** Enriched RNAs associated to OMVs were determined using *DESeq2*, calculating differential presence between intracellular and OMV-associated fractions for each condition. Expressed genes with an average number of reads over 5 for a given set of triplicates, a log_2_ Fold Change over 2, and a corresponding adjusted *p*-value under 0.05 were selected. The log_2_ Fold Change is displayed as colored squares from 2 (yellow) to 10 (dark blue).

Interestingly, a number of SPI-6 mRNAs were found to be exported and enriched in/on OMVs (**Figure 10**). In this case, an increased intracellular expression caused a greater export by vesicles, as it was clearly visible in Control conditions at high OD and SPI-1ind. The *in vitro* expression level of SPI-6 is generally low, as reported earlier (Mulder et al., 2012). However, OMV-association apparently occurred in all conditions, and to a higher extent in non-SPI-2ind conditions (**Figure 10B**). Interestingly, the same was observed concerning the enrichment in OMVs, showing a correlated expression and enrichment in vesicles for most of the SPI-6 transcribed RNAs (**Figure 10C** and **Supplementary Dataset F**). The coverage data of the mapped reads was relatively constant between intracellular and OMV-associated fractions (**Supplementary Figures S12A,B**).

SPI-6 in *S.* Typhimurium encodes for a Type 6 Secretion System (T6SS) that is involved in eukaryotic cell infection (Mulder et al., 2012). However, the role of the T6SS is not limited to virulence toward eukaryotes. Accumulating evidence demonstrates that the T6SS is mainly involved in interbacterial intoxication, targeting competitors by contact-dependent transport of bacteriocins from a donor cell to a recipient cell (Durand et al., 2014; Russell et al., 2014). Interestingly, one can note that the peptidoglycan hydrolase mRNA *tae4* (*STM0277*: validated as being present as a full-length transcript within OMVs: **Figure 5**) and its cognate immunity-protein mRNA *tai4* (*STM0278*) were only moderately present in the intracellular fraction (**Figure 10A**) under the conditions studied, but their mRNAs were enriched in/on OMVs purified in SPI-1ind and Control conditions at high OD (**Figure 10C**). Another pair of toxin/antitoxin encoded by the SPI-6 is *rhs* (*STM0291* and *STM0292*)/*rhsI* (*STM0293*). Rhs has a putative nuclease activity and was expressed in the intracellular case together with RhsI (**Figure 10A**). Both of their mRNAs were found associated with OMVs but only *rhs* was slightly enriched in several conditions (**Figures 10B,C**).

#### Some sRNAs Involved in Virulence Are Also Enriched in/on OMVs

**FIGURE 11:**
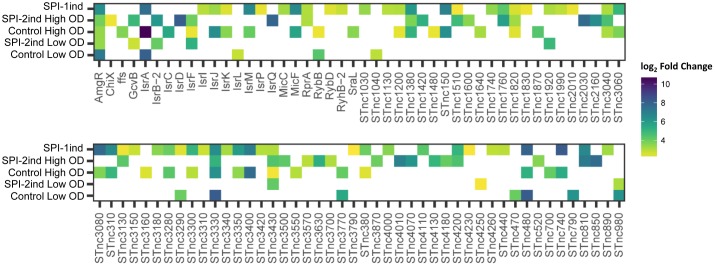
List of enriched sRNAs in/on OMVs isolated from the different culture conditions. For each enriched sRNA, the log_2_ Fold Enrichment in the OMV fraction is displayed as a colored square for the corresponding culture condition, from 2 (yellow) to 10 (dark blue). Enriched sRNAs associated to OMVs for each condition were determined using *DESeq2* (Love et al., 2014), calculating differential presence between intracellular and OMV-associated fractions for each condition. Transcripts with an average number of reads over 5 for a given set of triplicates, a log_2_ Fold Change over 2, and a corresponding adjusted *p*-value under 0.05 were selected.

As we could experimentally validate the presence of several full-length sRNAs (**Figure 5** and **Supplementary Dataset C**) and as we have seen in **Figure 8** that a smaller proportion of these latter was also enriched in/on OMVs in a condition-dependent manner, we analyzed in more detail these sRNAs (**Figure 11** and **Supplementary Dataset G**). The majority of them was found in the SPI-2ind and SPI-1ind cases, thus linked to virulence-like conditions. An enrichment in/on OMVs was mainly observed in high OD conditions; with only a few transcripts enriched at low OD. If these were exported in their native form through OMVs, they might be involved in pathway regulation in surrounding cells up-taking OMVs.

Traces of almost all pathogenicity-island encoded sRNA were found in OMVs (**Supplementary Figure S13**). Among them, one can note the presence of *IsrC*, triggering the degradation of the *mgrA* mRNA, involved in *Salmonella* survival in macrophages (Padalon-Brauch et al., 2008). It was expressed in our control (PCN) as reported earlier (Hébrard et al., 2012). We found that it was also enriched in/on OMVs generated in Control High OD condition, with a logFC = 5.45 (*p* < 0.001). No trace of it was found in/on OMVs from SPI-2ind conditions (**Supplementary Figure S13**). *IsrM* is known to be expressed in gut tract conditions *in vitro* (Hébrard et al., 2012). We found the transcript enriched in the control and SPI-1ind at high OD, but not in SPI-2ind. This sRNA is targeting *hilE* mRNA, modulating SPI-1 expression and repressing *sopA*. Δ*isrM* strains are less invasive and have a reduced growth rate in host cells or the gut tract (Hautefort et al., 2008; Gong et al., 2011). *IsrJ* is a *trans*-acting sRNA that was enriched in the control at high OD and SPI-1ind condition. It is upregulated in conditions which promote invasion of epithelial cells and affects the translocation efficiency of virulence-associated effector proteins into non-phagocytic cells (Padalon-Brauch et al., 2008).

Some core-genome sRNA were also found to be enriched. For example, *MicF* which is a Hfq-dependent *trans*-acting sRNA that regulates the synthesis of a lipid A modifying enzyme (Papenfort and Vogel, 2010) was enriched in SPI-1ind OMVs. Finally, *GcvB* was enriched in SPI-2ind conditions only, even if it was found associated to OMVs in all conditions. This sRNA is controlling amino-acids uptake and has also a demonstrated positive effect on bacteria resistance to low pH in *E. coli*, which is a physiological condition encountered by *Salmonella* in macrophages (Jin et al., 2009; Sharma et al., 2011).

Others sRNAs involved in virulence were also found in/on OMVs, but without specific enrichment regarding their intracellular presence. For example, *CrsB* and *CsrC* (the later validated experimentally to be internalized as a full-length transcript in OMVs by PCR: **Figures 5**, **6**, **Table 1**, and **Supplementary Dataset C**) were among the most exported sRNAs through OMVs, and the most abundant intracellular sRNAs (**Supplementary Figure S13**). These sRNAs control the translational regulator CsrA that regulates SPI-1 genes and the expression of flagella components, playing a central role in virulence (Fortune et al., 2006; Timmermans and Van Melderen, 2010). The 6S RNA, *SsrS* (validated experimentally to be internalized as a full-length transcript to OMVs by PCR: **Figures 5**, **6**, **Table 1**, and **Supplementary Dataset C**), was also highly expressed inside the cells and associated with OMVs. It is necessary for acidic-environment adaptation and epithelial cell invasion (Ren et al., 2017). *STnc1480* is a PhoP/Q and SlyA regulated sRNA whose role during the intracellular life of *Salmonella* has been suggested, and was found in OMVs in a high amount (Colgan et al., 2016). *SgrS*, a repressor of sugar uptake that also represses the SopD secreted virulence factor (Papenfort et al., 2012; Balasubramanian and Vanderpool, 2013), was also exported in all conditions.

## Discussion

This study provides a first global picture of the RNA cargo (>25 nt) associated with *S.* Typhimurium OMVs released under different *in vitro* culture conditions. These conditions mimic various environments encountered by *Salmonella* in its life cycle through the invasion and infection steps from the gut to macrophages. Even if this *in vitro* model does not fully reproduce the *in vivo* environment, a significant number of common pathways are induced, and among them a number of pathogenicity islands and virulence factors (Kröger et al., 2013; Srikumar et al., 2015). RNA was isolated from density gradient purified vesicles, with samples positive for a known outer membrane protein marker (OmpA), and lacking any obvious non-OMV structures like pili and flagellae, as judged by examination of TEM images (**Supplementary Figure S2**). These contaminating structures are present in crude OMV preparations (**Supplementary Figure S2B**). We cannot rule out the co-purification of nucleoprotein aggregates, however, these should be resistant to RNase A and the combination of RNase A and proteinase K. As the understanding of microbial membrane vesicles advances, we expect the validation of purity markers will provide the appropriate tools for even more stringent quality control.

The global quantity of OMVs and associated RNAs was not studied in this work. From western-blots, we could see differences in OmpA quantities between conditions. These do not automatically reflect differences in OMV secretion but can also originate from a change in the composition of the outer membrane. However, several studies already demonstrated the variation of membrane vesicles secretion induced by different cultivation conditions (Tashiro et al., 2010; McCaig et al., 2013). The global quantity of associated RNA depending on conditions did not clearly vary on our Bioanalyzer experiments, but quantification of such small amounts is challenging and should be addressed for further investigations (Aranda et al., 2009; Mateescu et al., 2017).

In accordance with several other studies, the majority of OMV-exported large RNAs were ribosomal RNAs (Dauros-Singorenko et al., 2018). It is interesting that these have been identified as a major immunostimulatory component for bacterial RNA recognition by dendritic cells and macrophages (Eberle et al., 2009; Li and Chen, 2012). They have also been reported to trigger a response from the human NLRP3 inflammasome in macrophages, as other types of RNAs do, thereby being part of the host–pathogen interactions during infection (Sha et al., 2014).

Other typically abundant ncRNAs, i.e., tRNAs were found associated with OMVs only at low levels in our data. This differs from previous observations on *E. coli* where they were among the most exported OMV-RNAs (Ghosal et al., 2015; Blenkiron et al., 2016). These differences might be due to the fact that we did not produce a small RNA sequencing library in the present study, which would probably better resolve the tRNA complement. As it has been demonstrated for *E. coli*, OMV-small-RNA libraries are highly enriched with fragmented tRNAs, largely at 15–50 nucleotides in length (Ghosal et al., 2015; Blenkiron et al., 2016).

In our data, the second most commonly found RNA type in OMVs were messenger RNAs, after rRNAs. The mRNA population included the majority of *Salmonella*’s expressed genes, however, there was a core population of transcripts that seemed always to be selected for export and enriched in the OMV RNA cargo. Some of them may originate from the OMV formation mechanism: one could expect some messengers coding for OMV-formation related proteins to be packaged at the same time as their product, as translation can occur in a spatial-dependent manner in bacteria (Nevo-Dinur et al., 2012; Buskila et al., 2014). However, RNA localization was also regulated independently from translation for some transcripts, suggesting a possible OMV-targeting process for specific mRNAs (Nevo-Dinur et al., 2011). Our data suggest that this differential packaging is adapted to environmental conditions and may be relevant for *Salmonella*’s virulence, as some populations of enriched mRNAs which were present in the RNA cargo were specific to each culture condition and contained pathogenicity-related mRNAs. This differs from previous findings in UPEC (Blenkiron et al., 2016). A common finding was the presence of prophage-originating RNAs, which are also known to represent pathogenicity-related regions. Type III secretion systems in *E. coli* are also connected to a vast network of phage-encoded genes which in turn are crucial for the evolution of pathogenesis (Tobe et al., 2006). This makes sense since phages are reported to be paramount in the evolution of bacterial pathogens by participating in horizontal gene transfer (Brussow et al., 2004). It is also important to note that the phage transcripts that we identified in our OMV RNA-Seq were encoded by temperate phages (phages that replicate using both lytic and lysogenic cycles) (Frye et al., 2005). We did not observe many bacterial cell lysis during our different culture conditions (**Supplementary Figure S1**), indicating that most of the phage-encoded RNA identified in our RNA-Seq data is originating from prophages. Nevertheless, we cannot exclude that the purification method used to purify OMVs did result in a co-purification of some active *Salmonella*’s phages, as gradient purification is sometimes also used for phage isolation (Effantin et al., 2010). As the phage capsid usually protects phage DNA from DNases and releases its content upon heating, “phage contamination” could also occur during cDNA library preparation (Owen et al., 2017). This should be verified in depth in upcoming studies as phage-related transcripts are enriched in/on OMVs purified in all tested conditions.

It is interesting to note that some OMV-associated mRNAs were enriched in/on vesicles compared to their intracellular fractions, suggesting that this export is not a passive but rather an active, regulated process. The purpose of such an operation and the underlying mechanism remain obscure. Read coverage of transcripts varied consequently depending on the exported RNA, sometimes showing an almost identical pattern to the coverage observed for the intracellular transcript, or on the contrary adopting distinct coverage profiles suggesting a specific processing or degradation. This dual nature was confirmed by PCR experiments in which we identified a number of native full-length RNAs associated with OMVs but failed to validate other predicted full-length transcripts. Whether this degradation or processing happens inside the cell or is ongoing during the long vesicle isolation procedure is an open question and further work should address this topic. However, we confirmed the presence of several full-length RNAs in independent replicates and by different techniques, showing that at least a fraction of RNAs are constantly associated and protected by OMVs.

Hence some OMV-associated RNAs might have a functional potential. This should be verified in future studies. Indeed, full-length mRNAs could potentially be translated by surrounding bacteria to which OMVs may be delivered. It could be a way to help members of the population by providing mRNAs even before a specific transcription activation signal has been received by the receptor cell, offering a quick response to a changing environment sensed by the exporting cell. RNA transfer between cells has been observed for exosome shuttles (Valadi et al., 2007). However, no comparable findings exist for OMV RNAs so far, even if evidence exists that OMV cargo in general is exchanged between bacteria [see for instance transfer of *Pseudomonas* Quinone Signal in *Pseudomonas aeruginosa*, (Mashburn and Whiteley, 2005)]. Bacterial exchange of OMVs is a preferential process, with different affinity and delivery efficiency for each receiving species (Tashiro et al., 2017). On the contrary, in the case of competing strains or host cells, these delivered mRNAs may “hack” the target’s translation machinery by producing virulence factors directly inside them, being part of the invasion toolkit of *Salmonella enterica*. In that sense, bacterial mRNA translation by mammalian cells has already been observed (Schoen et al., 2005). However, in this case prokaryotic RNAs were optimized for this purpose. For native mRNAs lacking eukaryotic nuclear processing like 5′ capping or the lack of internal ribosome entry sites (IRES), this process is likely to be impossible (Pelletier and Sonenberg, 1988; Pisarev et al., 2005).

sRNAs also populated the enriched transcripts which were associated with OMVs and their over-representation compared to the intracellular environment was mainly condition-dependent. However, it is difficult to see a relationship between the known implication of some sRNAs in virulence and the corresponding enrichment in vesicles. For instance, *STnc440*, *STnc470*, *STnc3750*, and *IsrH* are highly expressed in the *Salmonella* Containing Vacuole (Srikumar et al., 2015) but not specifically enriched in/on OMVs from SPI-2ind. A proportion of small RNAs originating from *E. coli* OMVs has been successfully aligned with the human genome in a way resembling eukaryotes’ long non-coding RNAs, which could also be the case in *Salmonella* with potential implications in host–pathogen communication (Celluzzi and Masotti, 2016). The interplay between the levels of regulatory sRNAs and their mRNA targets, together with their concomitant association to OMVs, would be an interesting point to look at in future work in order to be able to understand why given sRNAs are released *via* OMVs.

The confirmed presence of many full-length protein-associated RNAs in OMVs remains to be clarified. Whereas the export of SRP RNA might be explained by a side-effect of its location (proximity to the cytoplasmic membrane), the strong presence of others is mysterious. Interestingly, most of them are linked to virulence and resistance of *Salmonella* to external stresses to some extent. For instance, *10Sa* is involved in the regulation of specific genes linked to virulence and may take part in conferring resistance to the oxidative stress encountered throughout infection by preventing the buildup of abnormal proteins which may be toxic to the cell (Julio et al., 2000). Another interesting moiety is the 6S RNA (*SsrS*) which has been shown to be crucial for resistance and adaptation to acidic environment encountered during invasion (Ren et al., 2017). As we demonstrated that these molecules were inside OMVs rather than bound on the surface for *SsrS*, *CsrC*, *10Sa*, and *rnpB*, questions arise on the possible targeted mechanisms leading to packaging.

Finally, in OMVs isolated from *Borrelia burgdorferi* cultures, an enrichment of plasmid-encoded RNAs over chromosome-encoded transcripts has been recently shown (Malge et al., 2018). We validated the presence of a full-length plasmid-encoded transcript (pSLT035) in *Salmonella*-derived OMVs but in general we could not observe an enrichment of plasmid-encoded RNAs over chromosome-encoded transcripts associated with *Salmonella* OMVs (data not shown).

In summary, this work provides a first insight into the differential association of RNAs (>25 nt) with *Salmonella*-derived OMVs and emphasizes the impact of the bacteria-encountered environmental conditions on the RNA content of the vesicles. Much more work is needed to fully understand the underlying mechanisms and physiological processes that lead to this export, which results in the release of many virulence-associated RNAs and could have important consequences in host–pathogen interplay during infection. Whether this adaptable RNA export is a mechanism for targeted transfer or a way for cells to eliminate undesirable RNAs remains to be clarified. However, the confirmation of the export of full-length transcripts opens the door to numerous possible functional implications in cell-to-cell communication.

## Author Contributions

JF and PW conceived the original idea. JH, JF, and AM designed the experiments. JH, JF, AM, AH-B, JG, RH, AE, and DG performed the experiments. AH-B, PM, AM, and JF analyzed the data. AM and JF wrote the manuscript. All authors analyzed results, commented on and edited the manuscript.

## Conflict of Interest Statement

The authors declare that the research was conducted in the absence of any commercial or financial relationships that could be construed as a potential conflict of interest.
