# Supplementary material for: The RNA Complement of Outer Membrane Vesicles From Salmonella enterica Serovar Typhimurium Under Distinct Culture Conditions
Source: Front Microbiol. 2018 Aug 30;9:2015. doi: 10.3389/fmicb.2018.02015 (PMC6125333; doi:10.3389/fmicb.2018.02015)
Supplement: Supplementary file 2 [file Data_Sheet_1.PDF]

Figure S1

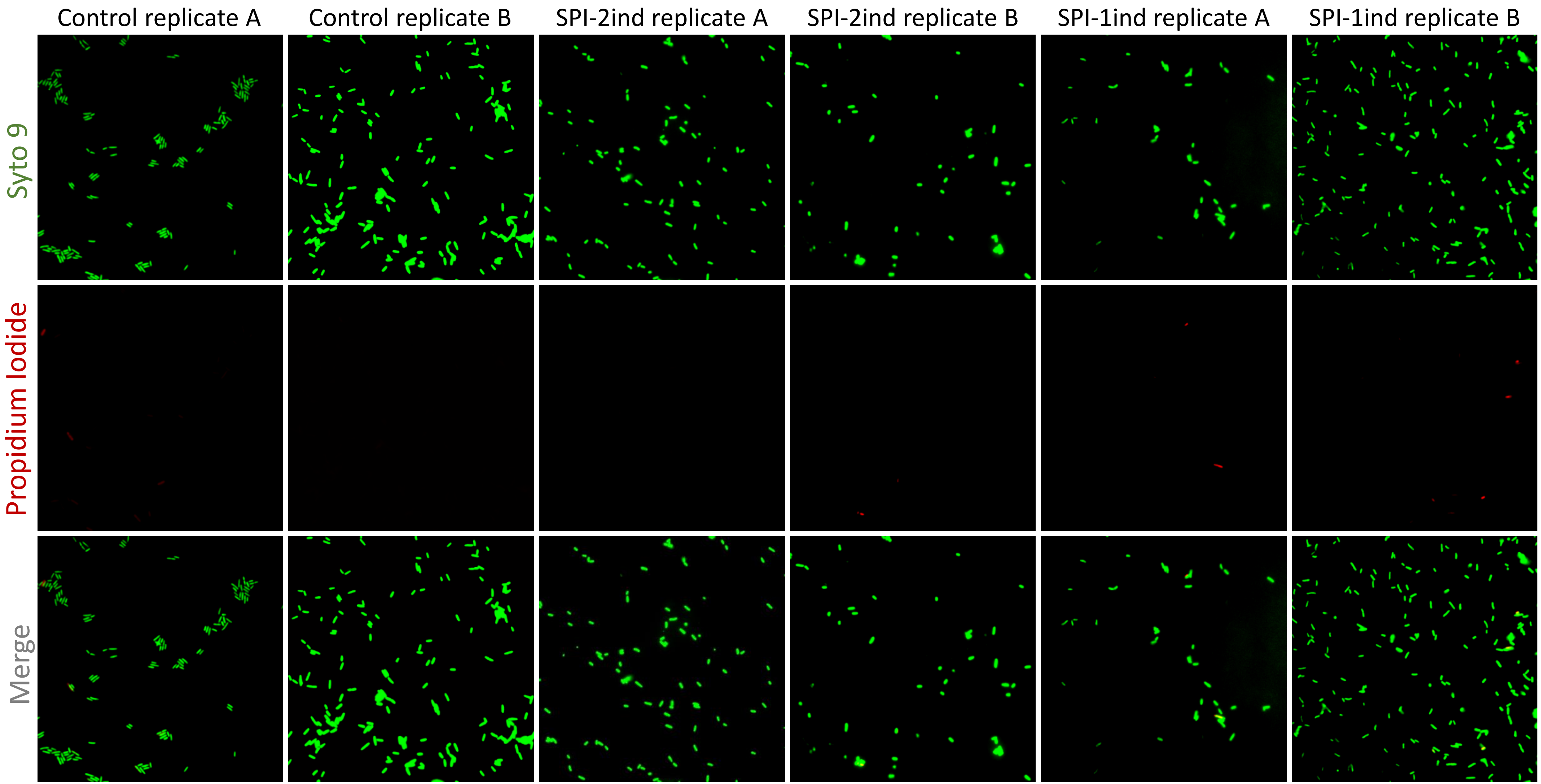

Control replicate A
Control replicate B
SPI-2ind replicate A
SPI-2ind replicate B
SPI-1ind replicate A
SPI-1ind replicate B
Syto 9
Propidium Iodide
Merge

Figure S1: Representative results of Live/Dead staining performed on bacterial cultures before OMV purification. All *Salmonella* cultures have been stained using LIVE/DEAD BacLight Bacterial Viability Kit according to manufacturer instructions (Life Technologies Europe BV, Belgium) and imaged using Microscope Axiovert 40CFL (Zeiss, Germany) with a 63 x magnification objective lens and Camera AxioCam MRn (Zeiss, Germany). Images were treated with ImageJ (Schneider et al., 2012). All bacterial cells are stained in green (Syto 9), and only dead cells are shown in red (Propidium Iodide). Thus, dead cells appear in yellow in the merged panels. These samples were obtained from High OD cultures used for RT-PCR.

Figure S2

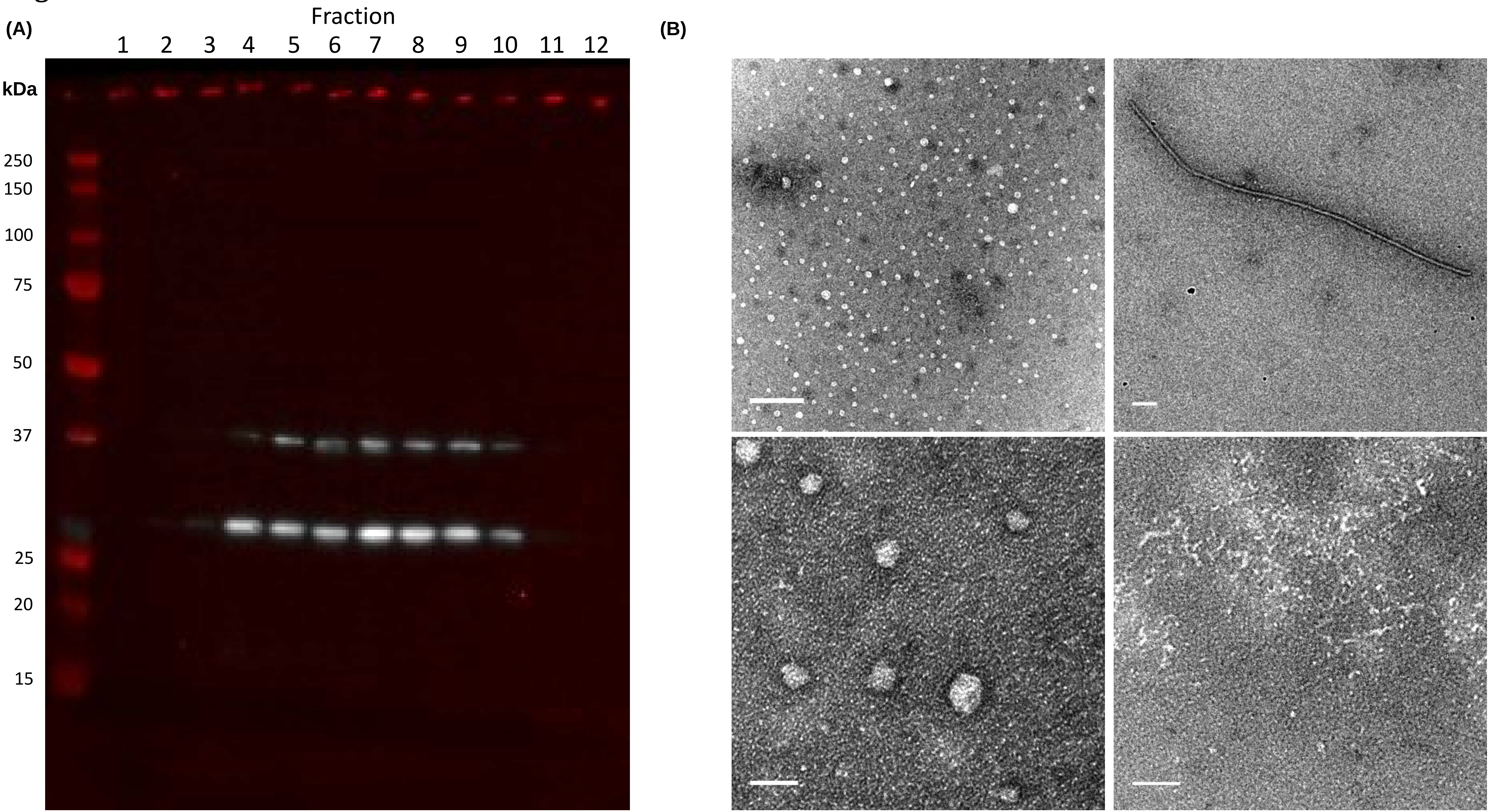

Figure S2

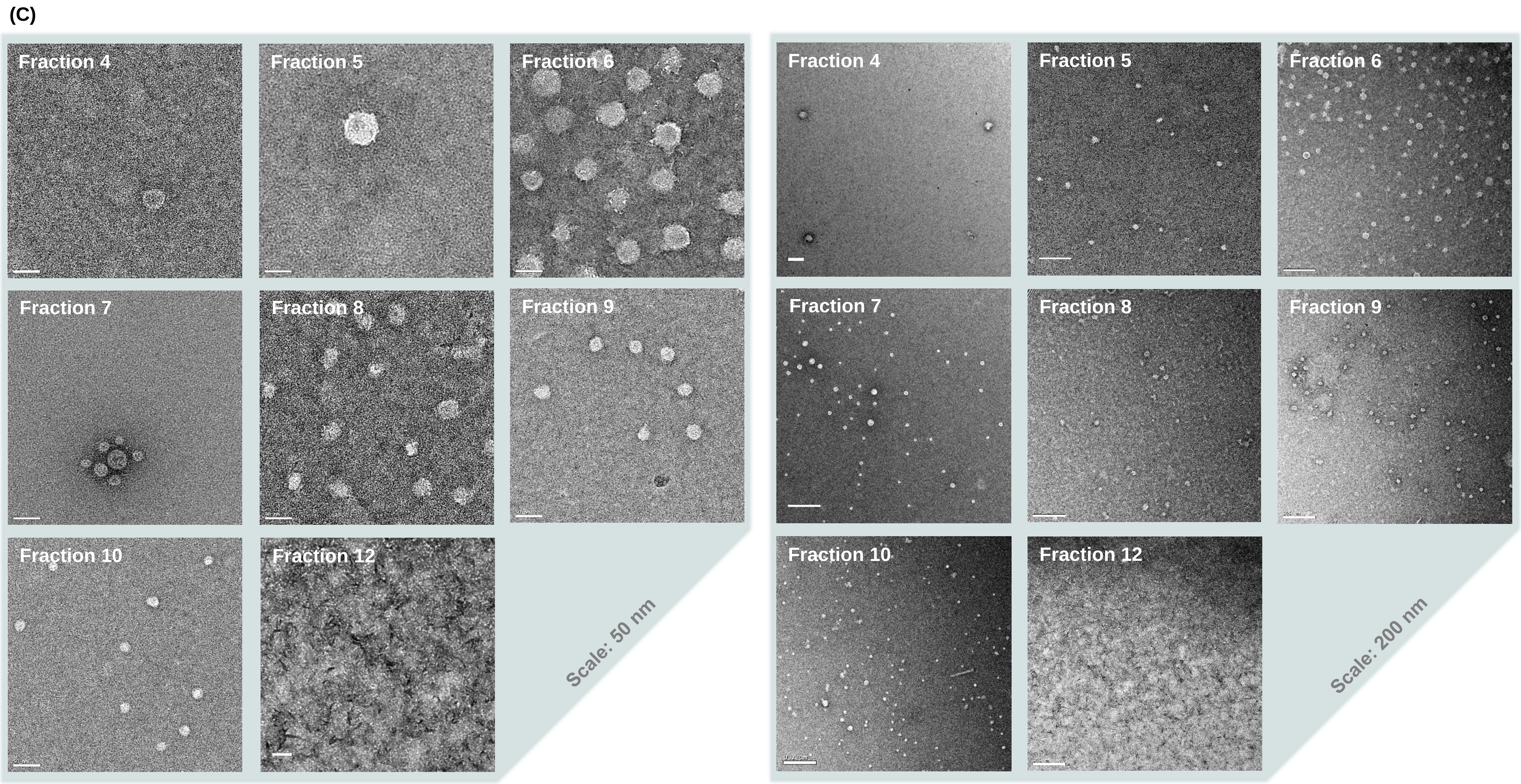
(C)
Fraction 4
Fraction 5
Fraction 6
Fraction 7
Fraction 8
Fraction 9
Fraction 10
Fraction 12
Scale: 50 nm
Fraction 4
Fraction 5
Fraction 6
Fraction 7
Fraction 8
Fraction 9
Fraction 10
Fraction 12
Scale: 200 nm

Figure S2: Combination of electron microscopy and SDS-PAGE to identify iodixanol gradient fractions containing pure *Salmonella*-derived OMVs. (A). 12.5 µl out of the 1 ml fractions of all the individual 12 iodixanol gradient fractions had been analyzed for the presence of *S*. Typhimurium outer membrane protein A (OmpA) by SDS-PAGE and western-blot. Fraction 1 being the lowest density fraction and fraction 12 being the highest density fraction. OmpA, an OMV-associated protein, is detected in fractions 4–10. The two bands are folded or unfolded proteins that do not migrate at the same speed (Kleinschmidt, Wiener, and Tamm 1999). **(B)**. Electron micrographs showing the composition of a crude OMV preparation before density gradient purification of the vesicles. Besides vesicles (left), large flagella fragments (upper-right) and protein aggregates (lower-right) are identified in the crude sample. Scale bar are 200 nm (top) or 50 nm (bottom). **(C)**. Electron micrographs showing that in fractions 4–10 spherical structures are present (OMVs have an average diameter of 20–200 nm and are bilayered). The densest fraction (12) contains thick aggregates that precipitate during the separation. Scale bars are 50 nm (left panel) or 200 nm (right panel).

Kleinschmidt, J. H., M. C. Wiener, and L. K. Tamm. 1999. “Outer Membrane Protein A of E. Coli Folds into Detergent Micelles, but Not in the Presence of Monomeric Detergent.” Protein Science : A Publication of the Protein Society 8 (10): 2065–71.

Figure S3

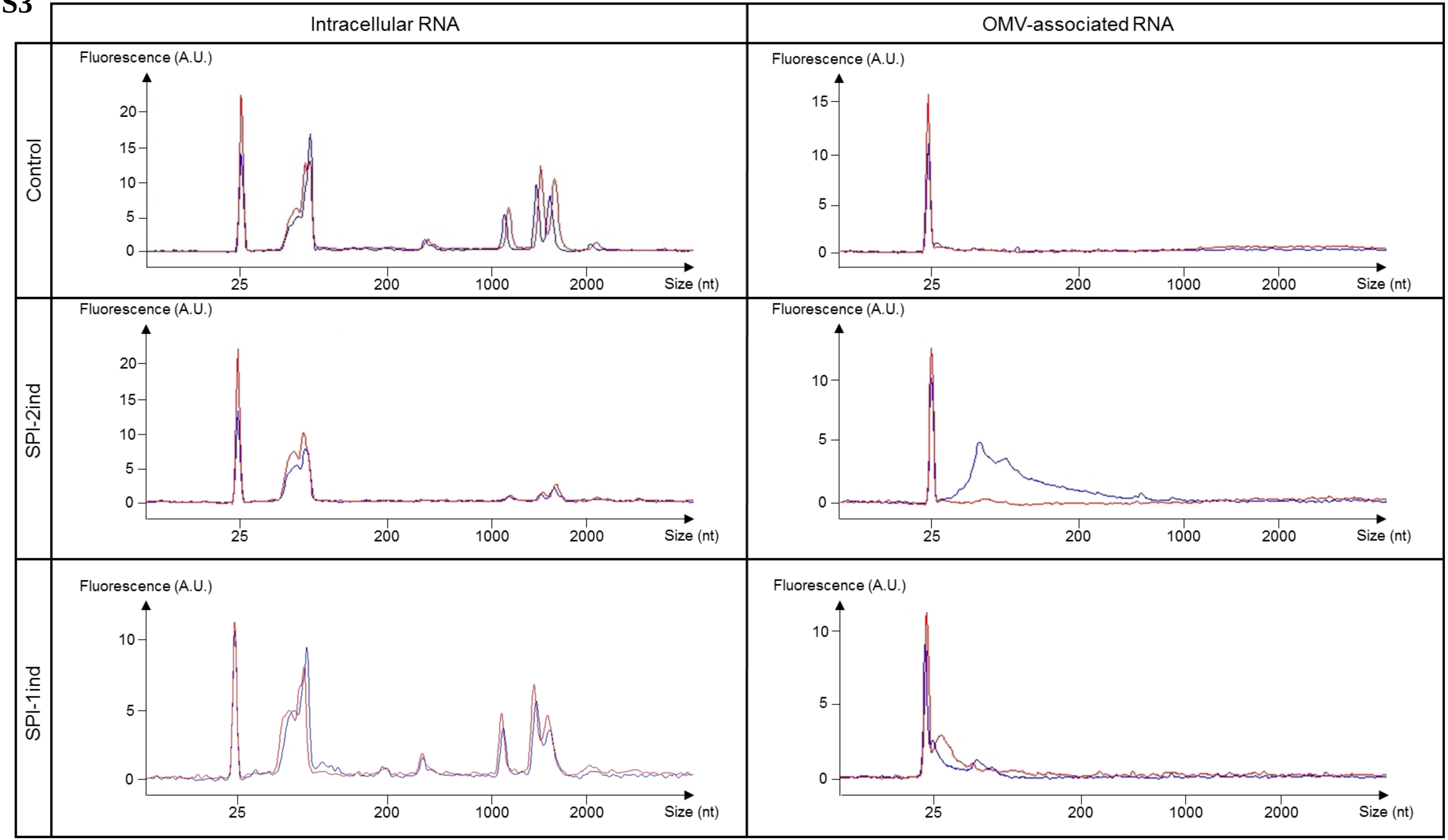
Intracellular RNA
OMV-associated RNA
Control
SPI-2ind
SPI-1ind
Fluorescence (A.U.)
Size (nt)
0
5
10
15
20
25
200
1000
2000

Figure S3: RNA size repartition from *Salmonella*-derived intracellular or OMV-associated RNA samples. 1 µl of extracted RNA from intracellular (diluted 1000x) and OMV extracts (no dilution, corresponding to 0.75 l of culture) was quality-checked with an Agilent 2100 Bioanalyzer. A pico RNA chip has been used to detect transcripts above 25 nucleotides. The 25 nucleotides band is due to an internal marker. The three major peaks between 1000 and 2000 nucleotides on intracellular graphs correspond to the three versions of *Salmonella* LT2's strain 23S ribosomal RNAs (Mattatall and Sanderson, 1996). Graphs from OMV-associated samples show that a small-RNA peak is sometimes visible, even if many RNAs were sequenced and validated by PCR on samples without a peak (see main text). Red and blue lines represent two biological replicates. A.U. : arbitrary unit. Samples represented here were obtained from cultures used for RT-PCR, where total RNA had been extracted using TRIzol reagent without any RNA size cut-off.

Mattatall, N.R., Sanderson, K.E., 1996. *Salmonella* typhimurium LT2 possesses three distinct 23S rRNA intervening sequences. J. Bacteriol. 178, 2272–2278.

Figure S4

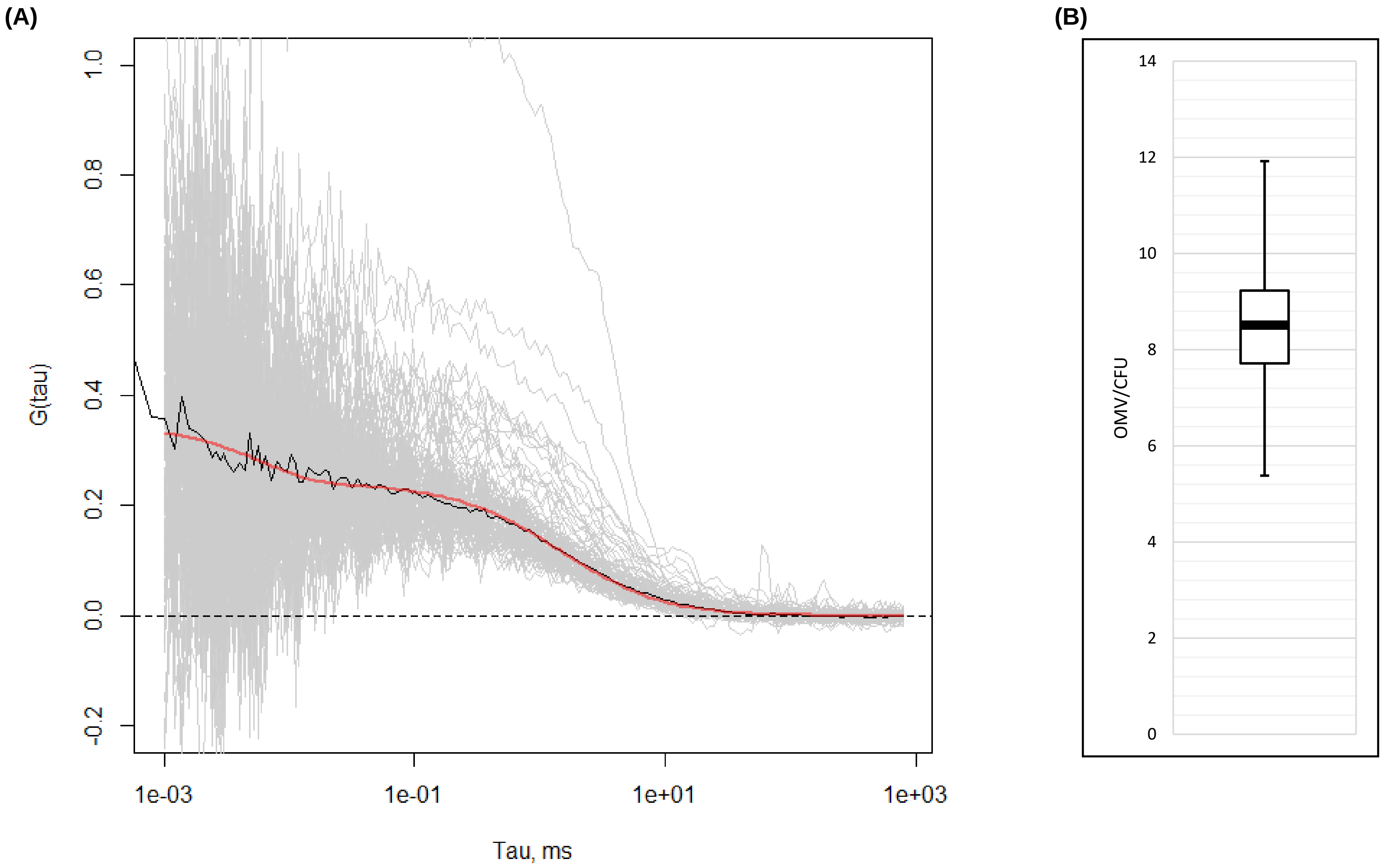

(A)
G(tau)
1.0
0.8
0.6
0.4
0.2
0.0
-0.2
1e-03
1e-01
1e+01
1e+03
Tau, ms
(B)
OMV/CFU
14
12
10
8
6
4
2
0

Figure S4: Estimation of OMV secretion by Fluorescence Correlation Spectroscopy.

OMVs obtained from SPI-1ind condition, concentrated by tangential flow, were stained with 100 nM of Nile Red lipophilic fluorescent dye (Sigma Aldrich). **(A)**. FCS autocorrelation curves $G(\tau)$ (grey lines, n=100) and the corresponding median curve (black line) retrieved using a two-photon excitation platform (see Materials and Methods). Number of diffusing species and diffusion parameters were retrieved by adjusting curves with Equation 1 (red line) **(B)**. Boxplot showing the median, first and third quartiles, minimum and maximum values of the number of OMV amount deduced from (A). OMV concentration was normalized to the number of Colony Forming Units in the culture and corresponds to 0.043 nM.

Figure S5

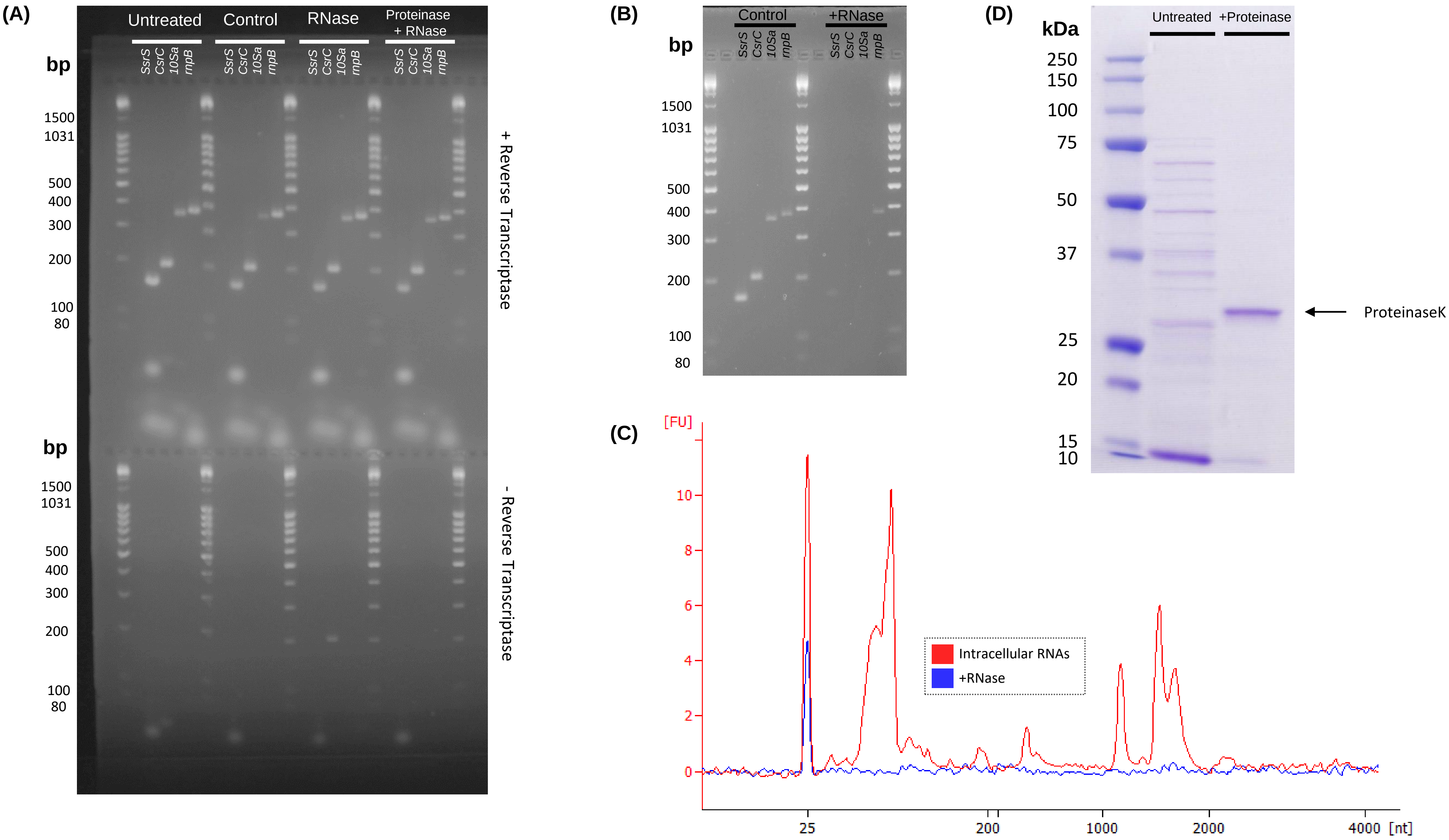

(A)
Untreated
Control
RNase
Proteinase + RNase
SsrS CsrC 10Sa rnpB
bp
1500
1031
500
400
300
200
100
80
+ Reverse Transcriptase
- Reverse Transcriptase
(B)
Control
+RNase
(C)
[FU]
Intracellular RNAs
+RNase
25
200
1000
2000
4000 [nt]
(D)
kDa
Untreated
+Proteinase
250
150
100
75
50
37
25
20
15
10
ProteinaseK

Figure S5: RNase protection assay of selected protein-associated sRNAs isolated in OMVs. (A) OMV isolation was performed from SPI-1ind condition as described in Materials and Methods, except that a supplemental enzymatic step was added before density gradient ultracentrifugation. Crude OMV preparation was divided in four fractions. One was kept untouched (Untreated). The second was subjected to the same protocol than the third and fourth fraction without any enzyme added (Control). The third was digested by RNaseA. The fourth was digested by ProteinaseK followed by proteinase inactivation and RNAseA digestion. The protocol was then followed as described in Materials and Methods until cDNA libraries from OMV-associated RNAs were obtained. End-point PCR was then conducted with the same primers and conditions used previously, for *SsrS*, *CsrC*, *10Sa* and *rnpB* genes. Resulting samples were deposited on a 3% w/v agarose gel in TBE buffer, subjected to electrophoresis and stained with Ethidium Bromide. +RT samples are shown on the top gels, and each corresponding –RT control is visualized on the lower row of gels. **(B)** Positive control of RNA degradation by RNaseA treatment. 10 ng of intracellular RNA extracted from *Salmonella* grown in SPI-1ind condition were digested as described in Materials and Methods before cDNA synthesis. PCR and electrophoresis were done in the same conditions than in (A). Only *rnpB* was still detected by RT-PCR after treatment. **(C)** Bioanalyzer run of untreated and RNase treated intracellular RNAs obtained in (B). **(D)** Positive control of protein degradation by proteinaseK treatment. 5 µg of intracellular proteins extracted from *Salmonella* grown in SPI-1ind condition were digested as described in Materials and Methods, and then subjected to SDS-PAGE using a 12% Bis-Tris Criterion TM XT precast gel (Biorad) in MOPS buffer. Revelation was achieved with Imperial protein stain (Thermo Scientific).

Figure S6

(A) Exported RNAs

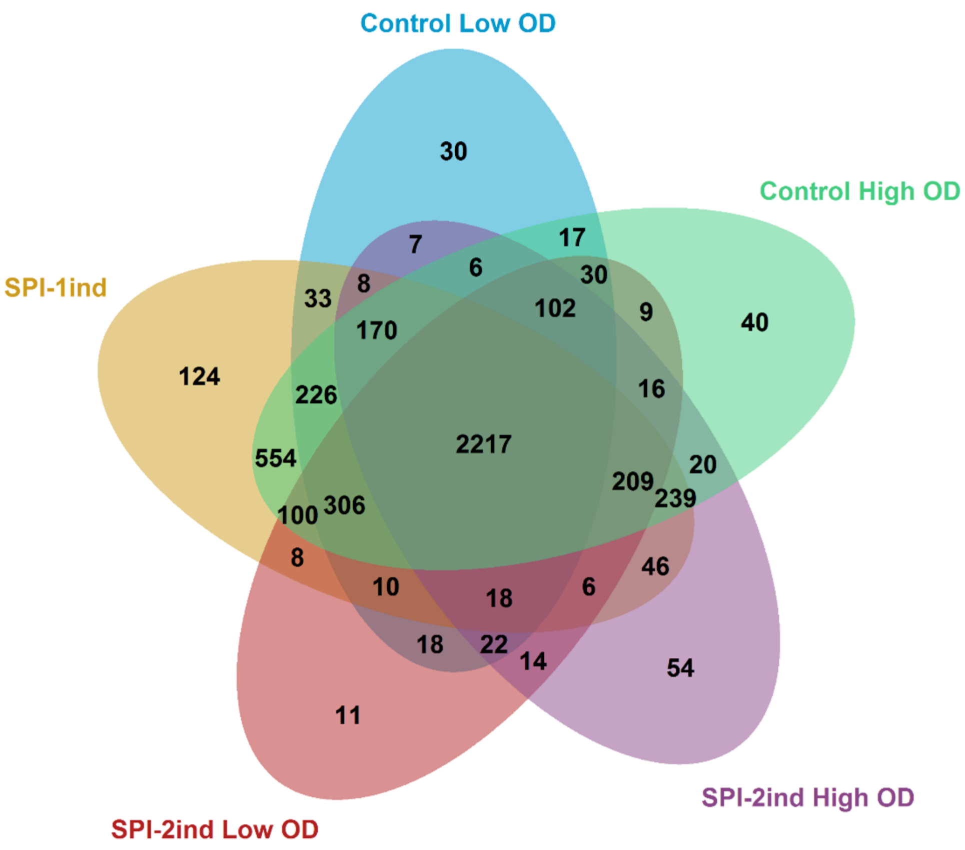

(B) Enriched RNAs

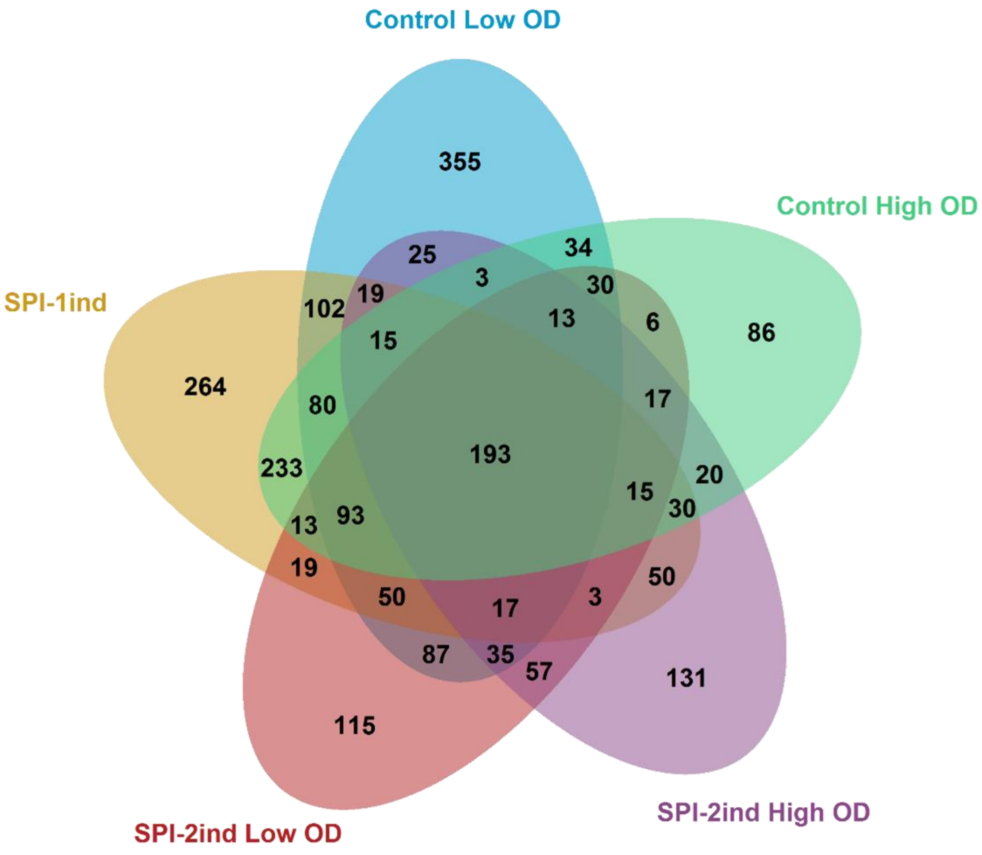

Figure S6: Comparison of exported and enriched RNAs in OMVs isolated from different *in vitro* conditions. (A). Venn diagram representing the number of OMV-associated RNAs shared between different conditions. The counts of OMV-associated RNAs were normalized using *DESeq2* and filtered for a minimum averaged count of 5 reads per biological triplicate. One unit represents one expressed gene. **(B)**. Venn diagram representing the number of enriched RNAs sequenced in the OMV fractions, shared between different conditions. One unit represents one expressed gene. Enriched RNAs were determined using *DESeq2*, calculating differential presence between intracellular and OMV-associated fractions for each condition. Expressed genes with an average number of reads over 5 for a given set of triplicates, a $\log_2$ Fold Change over 2, and a corresponding adjusted p-value under 0.05 were selected. Identical datasets were used for the generation of Figure 8 and Figure S6.B.

Figure S7

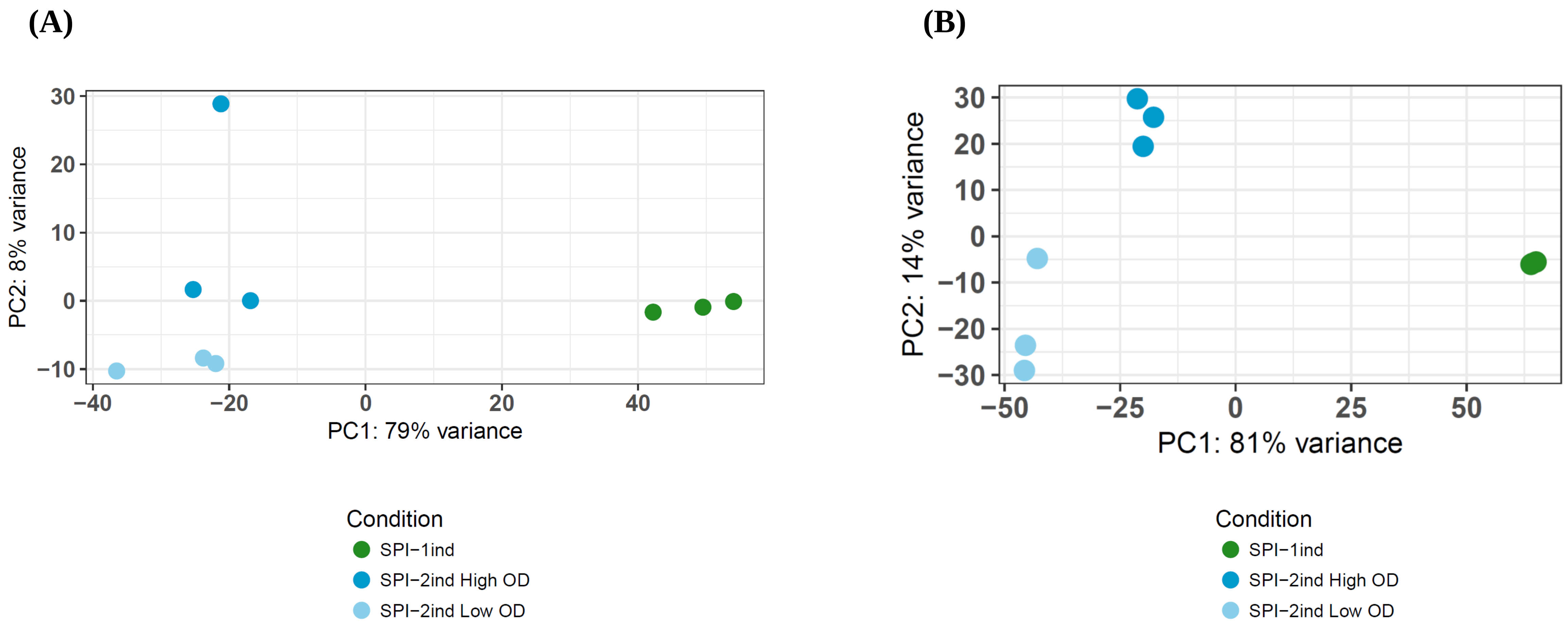
(A)
PC2: 8% variance
PC1: 79% variance
30
20
10
0
−10
−40
−20
0
20
40
Condition
SPI−1ind
SPI−2ind High OD
SPI−2ind Low OD
(B)
PC2: 14% variance
PC1: 81% variance
30
20
10
0
−10
−20
−30
−50
−25
0
25
50
Condition
SPI−1ind
SPI−2ind High OD
SPI−2ind Low OD

Figure S7.A-B: Principal Component Analyses. (A). OMV-associated samples in SPI-1ind and SPI-2ind conditions, including rRNA. **(B).** Intracellular samples in SPI-1ind and SPI-2ind conditions, omitting rRNA. Normalized counts obtained with the *DESeq2* package were used as input.

Figure S8.A

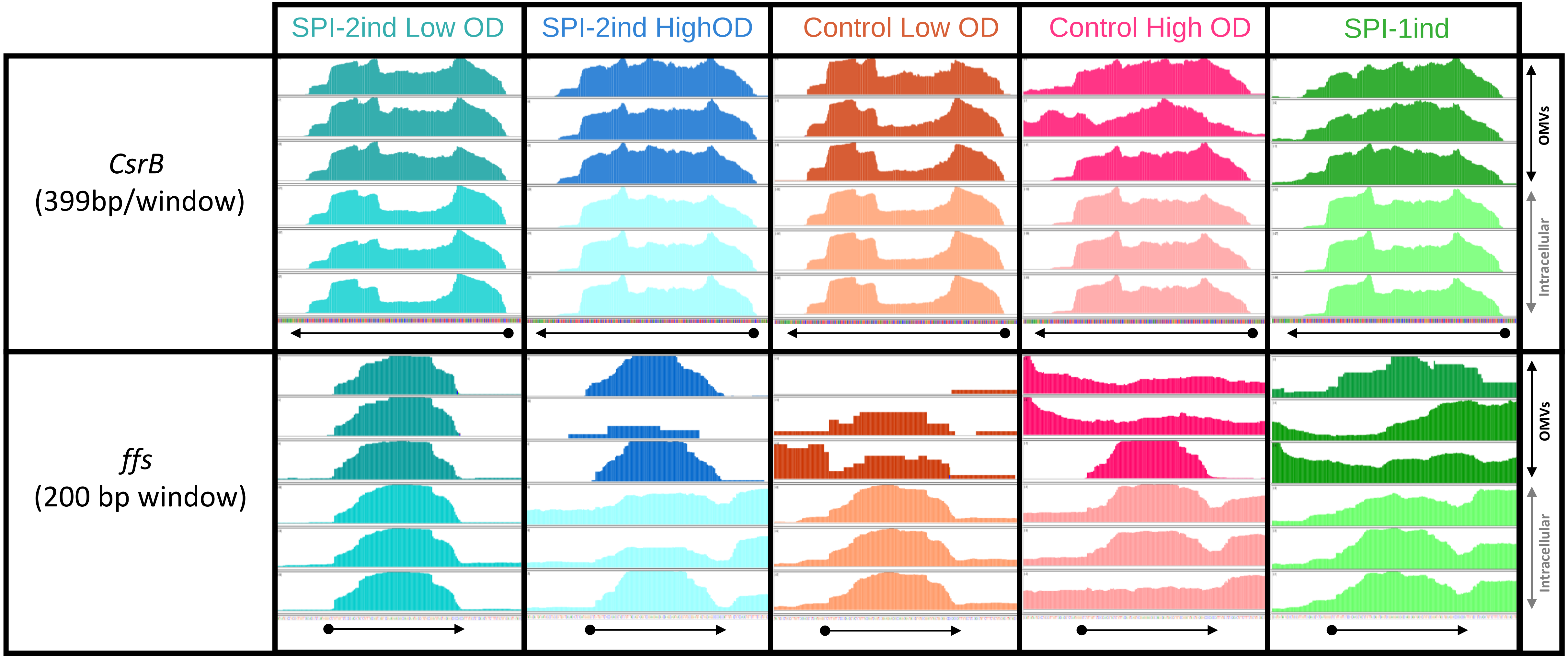
SPI-2ind Low OD
SPI-2ind HighOD
Control Low OD
Control High OD
SPI-1ind
CsrB
(399bp/window)
ffs
(200 bp window)
OMVs
Intracellular
OMVs
Intracellular

Figure S8.B

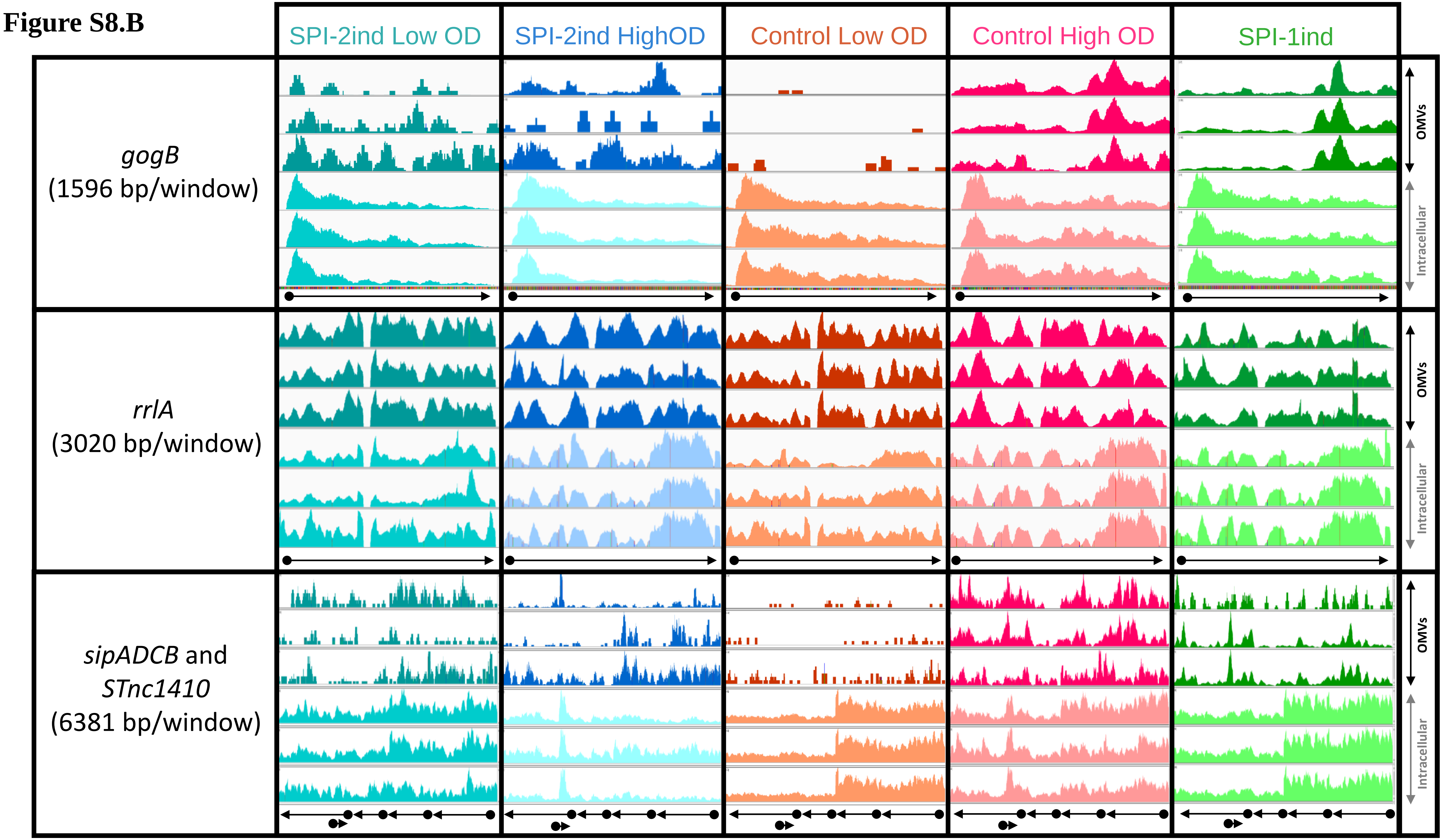

SPI-2ind Low OD
SPI-2ind HighOD
Control Low OD
Control High OD
SPI-1ind
gogB
(1596 bp/window)
rrlA
(3020 bp/window)
sipADCB and
STnc1410
(6381 bp/window)
OMVs
Intracellular

Figure S8.C

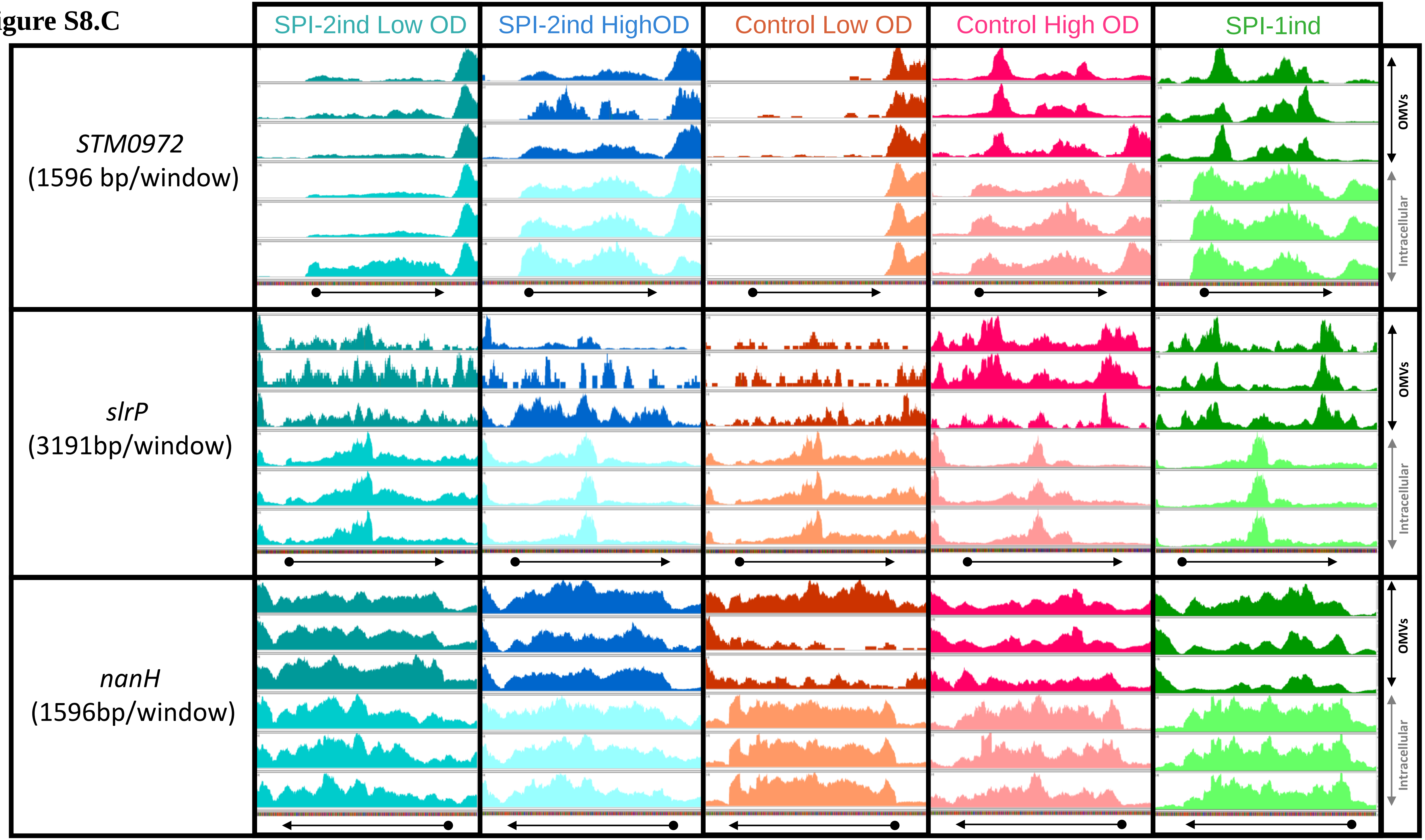
SPI-2ind Low OD
SPI-2ind HighOD
Control Low OD
Control High OD
SPI-1ind
STM0972
(1596 bp/window)
slrP
(3191bp/window)
nanH
(1596bp/window)
OMVs
Intracellular

Figure S8.A-C: Visualization of various intracellular and OMV-related read coverage plots of distinct genes in distinct culture conditions. RNA coverage from sequencing data was visualized with the Integrative Genomics Viewer software (v2.4.8) using default parameters (Robinson et al., 2011). Each plot represents the raw number of reads mapped along the observed sequence, automatically scaled relatively to the highest number of read existing in this portion of the genome. The three plots in dark colors at the top of each window represents the sequencing coverage of OMV-associated fractions, in biological triplicate for each condition. On the contrary, the three plots in lighter colors at the bottom of each window show the coverage for the same RNA but from the corresponding intracellular fractions. The arrows under each window precise the genes position and orientation, according to the data extracted from the *Salmonella* LT2 genome annotation (NCBI accession number AE006468.2) or pSLT plasmid annotation (NCBI accession number AE006471.2). For each gene, the size of the observed portion of the genome is precised on the left. One can appreciate the relative identical coverage between intracellular and extracellular fractions for some transcripts (*CsrB*) or drastic changes between the relative repartitions of reads (*STM0972, slrP*).

Figure S9

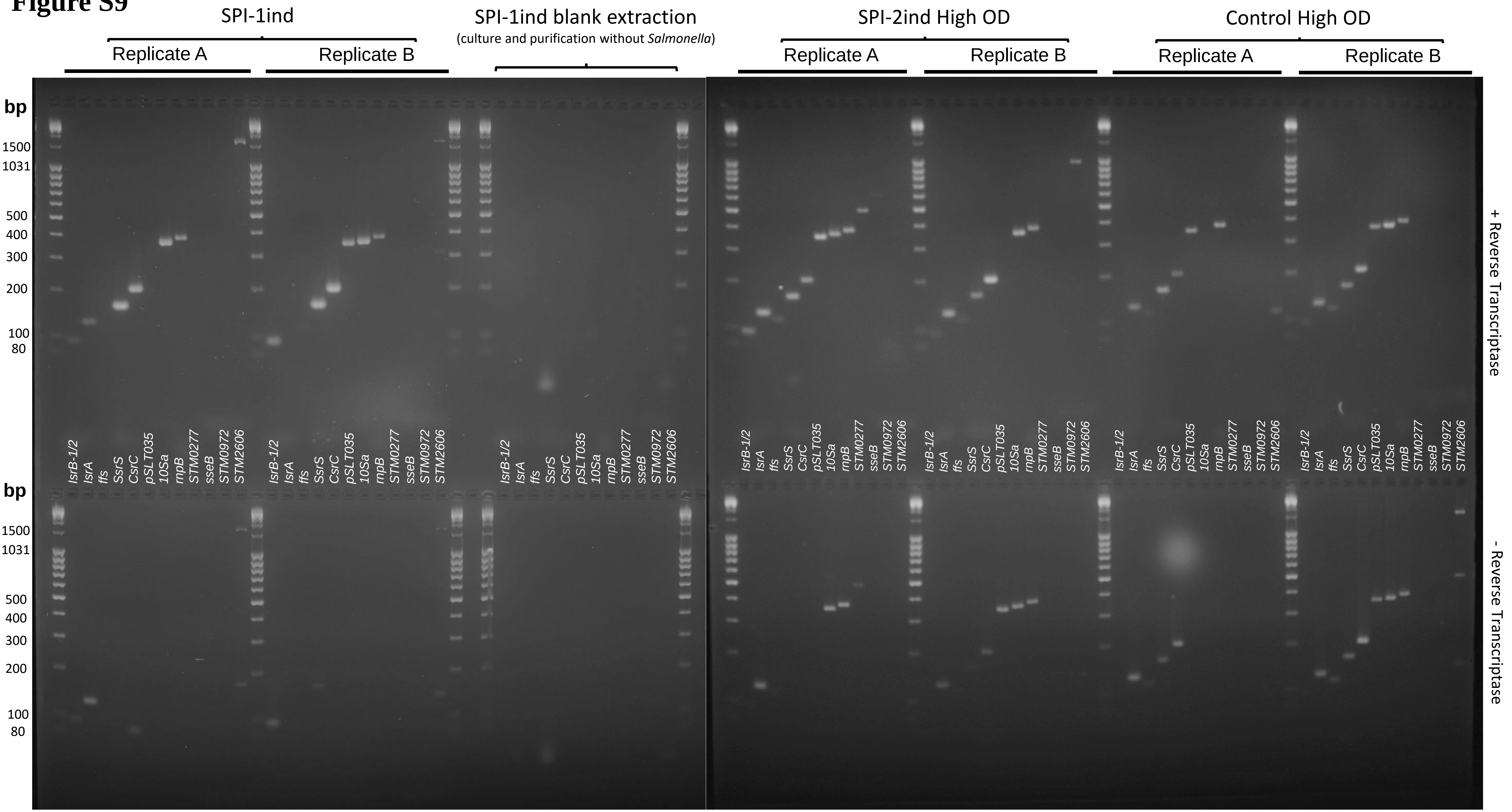

SPI-1ind
SPI-1ind blank extraction
(culture and purification without *Salmonella*)
SPI-2ind High OD
Control High OD
Replicate A
Replicate B
Replicate A
Replicate B
Replicate A
Replicate B
bp
1500
1031
500
400
300
200
100
80
IsrB-1/2
IsrA
ffs
SsrS
CsrC
pSLT035
10Sa
rnpB
STM0277
sseB
STM0972
STM2606
+ Reverse Transcriptase
- Reverse Transcriptase

Figure S9: Agarose gel visualization of selected OMV-associated RNAs that were amplified in their full-length by RT-PCR. After OMVs isolation and RNA extraction as described in Materials and Methods, cDNA synthesis was performed. End-point PCR was then conducted with primers flanking the coding sequence (mRNA) or the full sequence (sRNA) of 30 selected RNAs. Resulting samples were deposited on a 3% w/v agarose gel in TBE buffer, subjected to electrophoresis and stained with Ethidium Bromide. Only transcripts that were amplified as full-length amplicons are shown (12 out of 30 selected RNAs that were tested).Two biological replicates for SPI-1ind, SPI-2ind High OD and Control High OD conditions are visualized. Moreover, when the same protocol was performed on an identical culture without inoculation, described here as SPI-1ind blank extraction, no amplification for any of the tested genes was observed. +RT samples are shown on the top row of gels, and each corresponding –RT control is visualized on the lower row of gels. Samples were deposited in between two ladders as follows (from left to right): *IsrB-1/2, IsrA, ffs, SsrS, CsrC, pSLT035, 10Sa, rnpB, STM0277, sseB, STM0972, STM2606.*

Figure S10

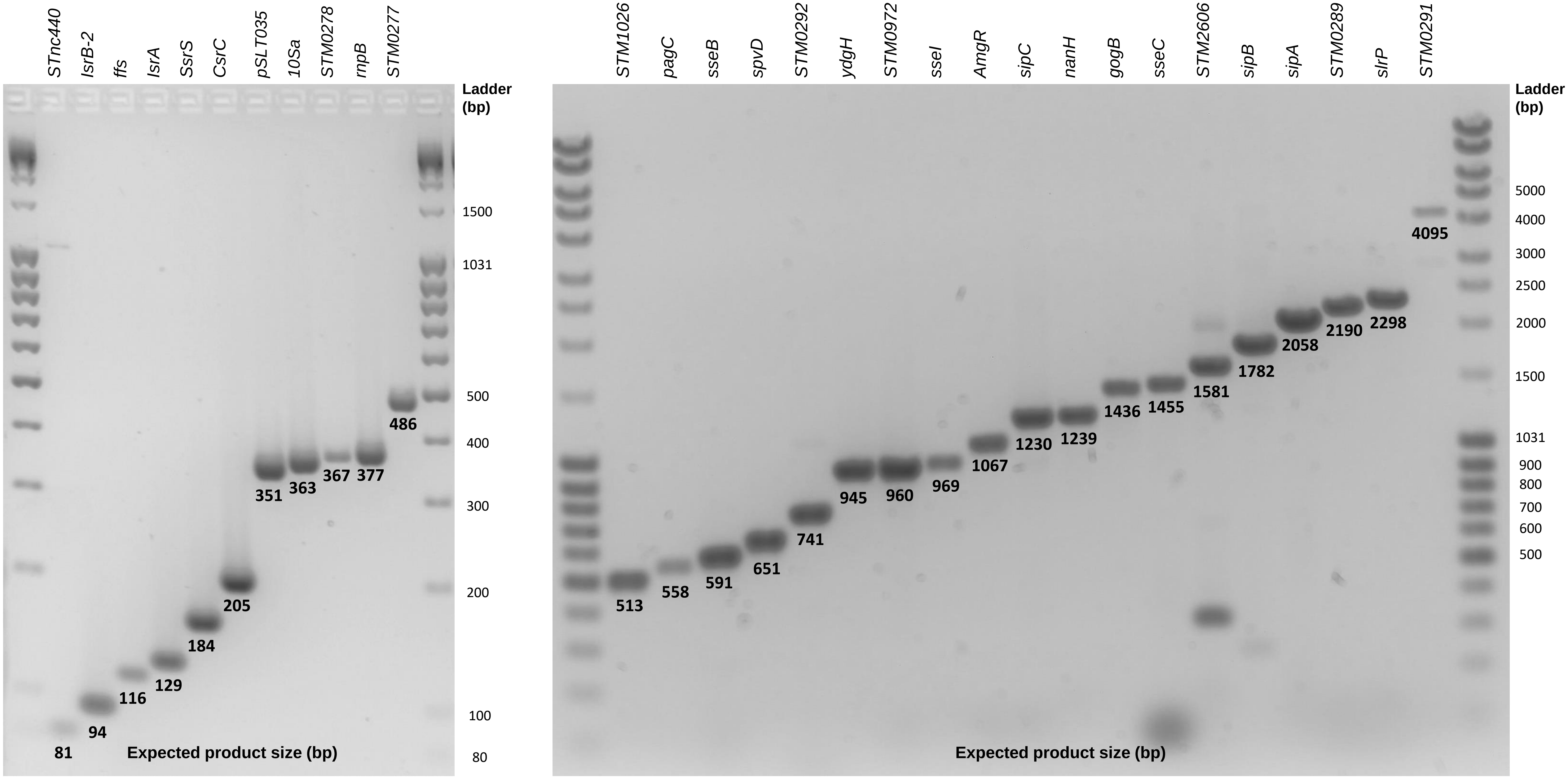

STnc440
IsrB-2
ffs
IsrA
SsrS
CsrC
pSLT035
10Sa
STM0278
rnpB
STM0277
Ladder (bp)
1500
1031
500
400
300
200
100
80
81
94
116
129
184
205
351
363
367
377
486
Expected product size (bp)
STM1026
pagC
sseB
spvD
STM0292
ydgH
STM0972
sseI
AmgR
sipC
nanH
gogB
sseC
STM2606
sipB
sipA
STM0289
slrP
STM0291
Ladder (bp)
5000
4000
3000
2500
2000
1500
1031
900
800
700
600
500
513
558
591
651
741
945
960
969
1067
1230
1239
1436
1455
1581
1782
2058
2190
2298
4095
Expected product size (bp)

Figure S10: Agarose gel visualization of intracellularly expressed full-length RNAs by RT-PCR. Amplification of intracellularly expressed full-length transcripts were used as positive controls, in order to validate PCR primers and protocols. After cell lysis and RNA extraction from SPI-1ind condition as described in Materials and Methods, cDNA synthesis was performed. End-point PCR was then conducted with primers flanking the coding sequence (mRNA) or the full sequence (sRNA) of 30 selected RNAs. Resulting samples were deposited on a 3% w/v agarose gel in TBE buffer (left), or on a 1% w/v agarose gel in TAE buffer (right) depending on their size, and subjected to electrophoresis before being stained with Ethidium Bromide.

Figure S11.A

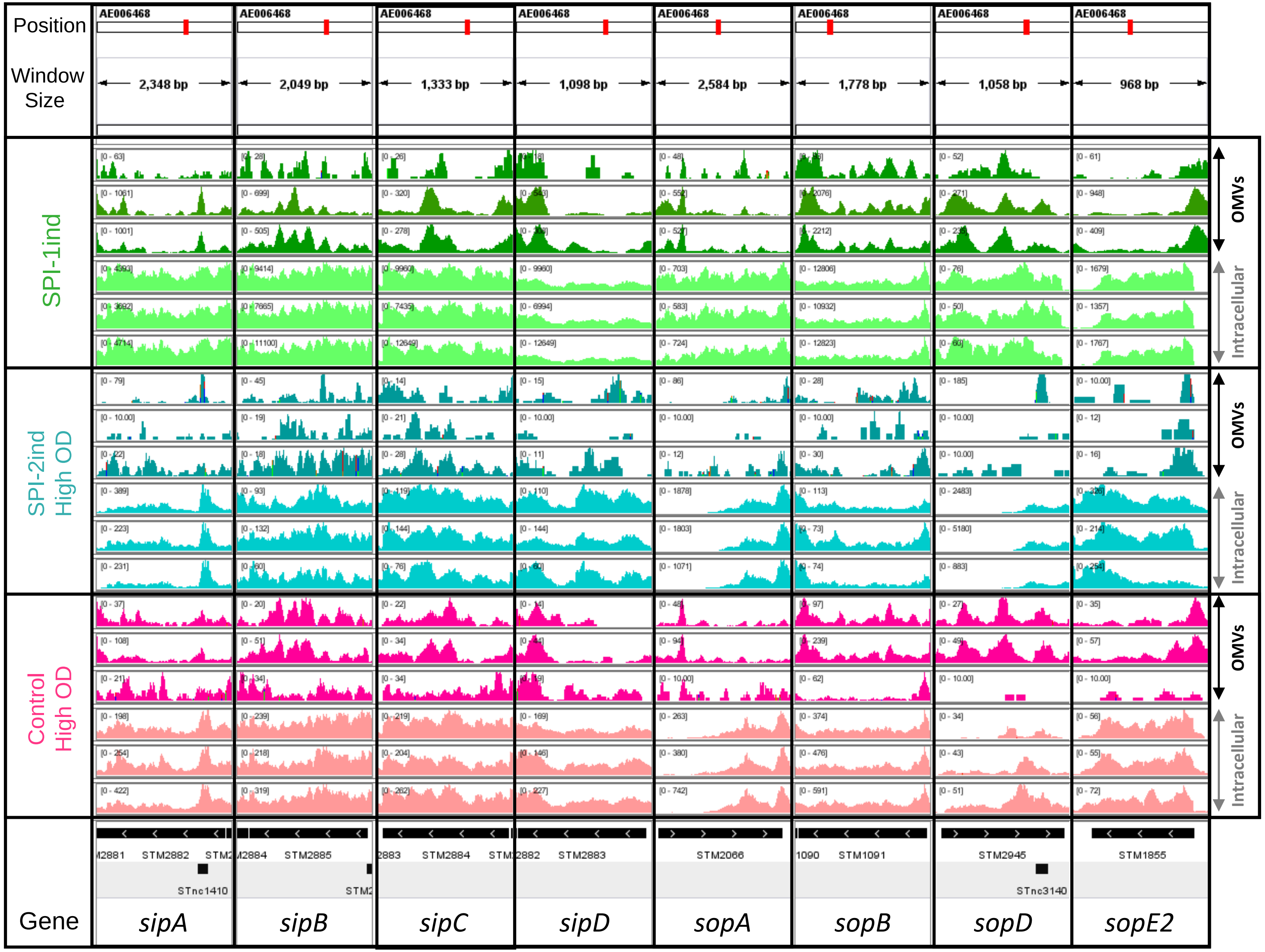
Position
Window Size
AE006468
2,348 bp
2,049 bp
1,333 bp
1,098 bp
2,584 bp
1,778 bp
1,058 bp
968 bp
SPI-1ind
SPI-2ind High OD
Control High OD
OMVs
Intracellular
STM2882
STM2885
STM2884
STM2883
STM2066
STM1091
STM2945
STM1855
STnc1410
STnc3140
Gene
sipA
sipB
sipC
sipD
sopA
sopB
sopD
sopE2

Figure S11.B

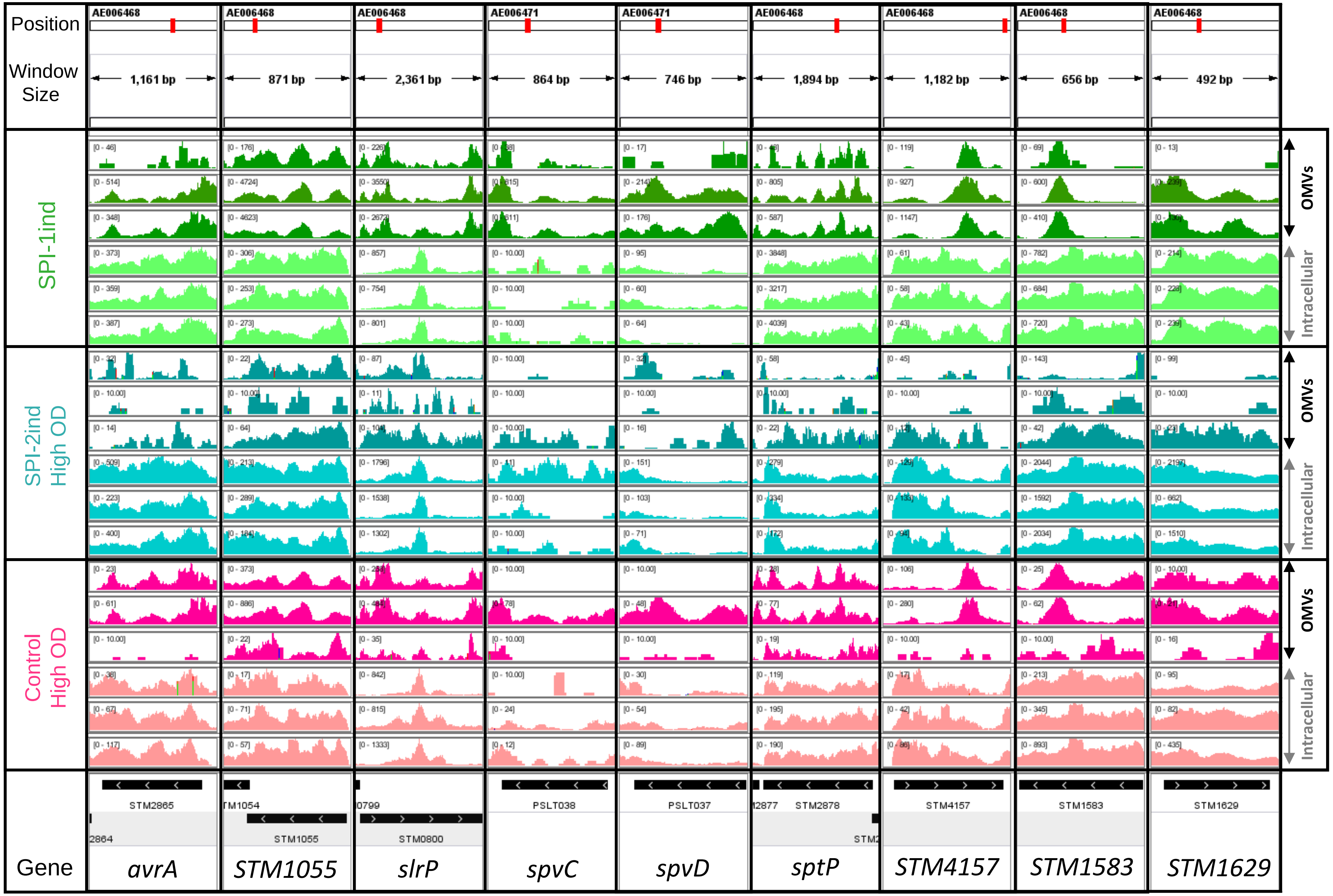

Position
Window Size
AE006468
AE006471
1,161 bp
871 bp
2,361 bp
864 bp
746 bp
1,894 bp
1,182 bp
656 bp
492 bp
SPI-1ind
SPI-2ind High OD
Control High OD
OMVs
Intracellular
Gene
STM2865
STM1055
STM0800
PSLT038
PSLT037
STM2878
STM4157
STM1583
STM1629
avrA
STM1055
slrP
spvC
spvD
sptP
STM4157
STM1583
STM1629

Figure S11.C

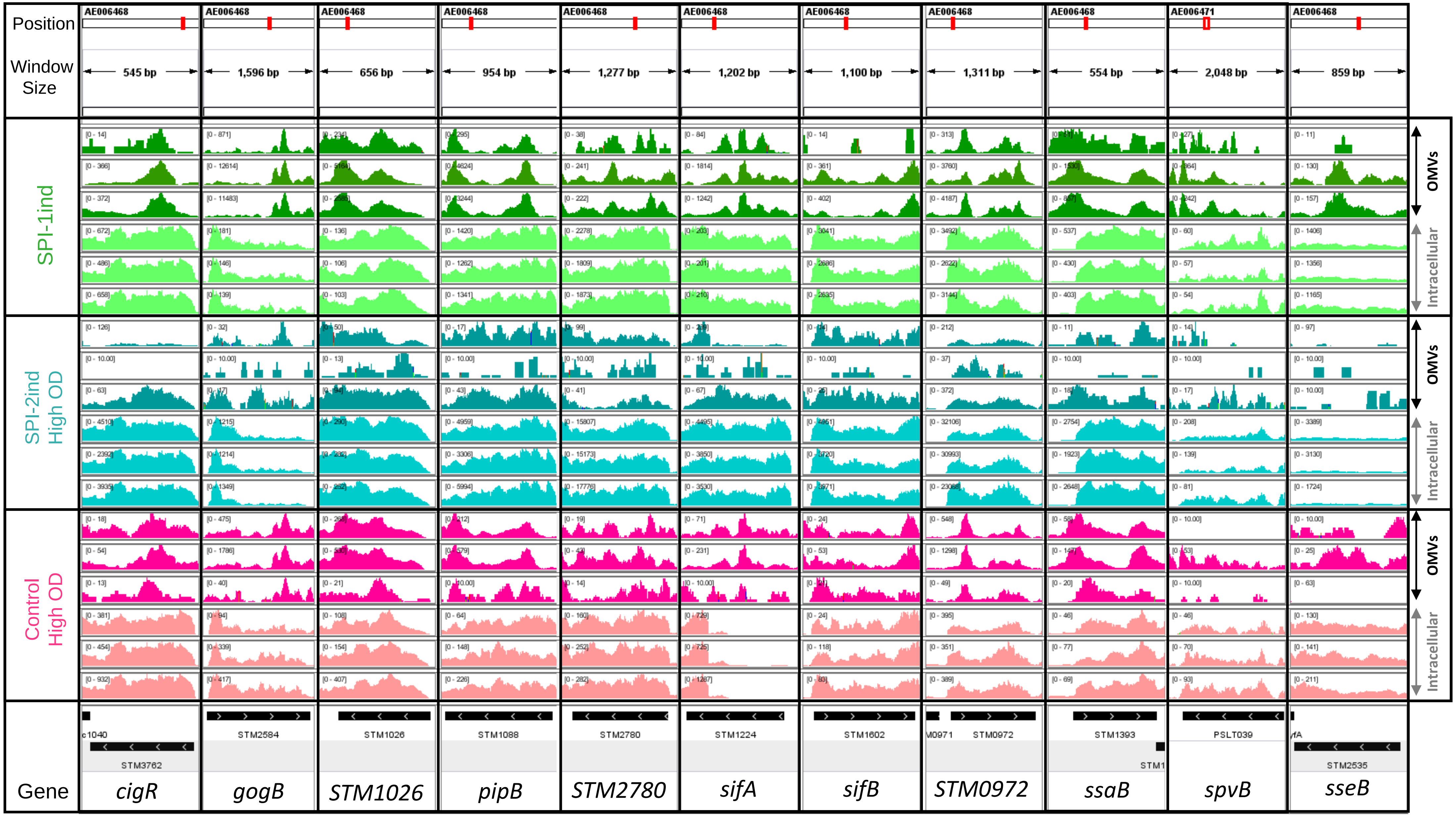

Position
Window Size
AE006468
AE006471
545 bp
1,596 bp
656 bp
954 bp
1,277 bp
1,202 bp
1,100 bp
1,311 bp
554 bp
2,048 bp
859 bp
SPI-1ind
SPI-2ind High OD
Control High OD
OMVs
Intracellular
Gene
cigR
gogB
STM1026
pipB
STM2780
sifA
sifB
STM0972
ssaB
spvB
sseB

Figure S11.D

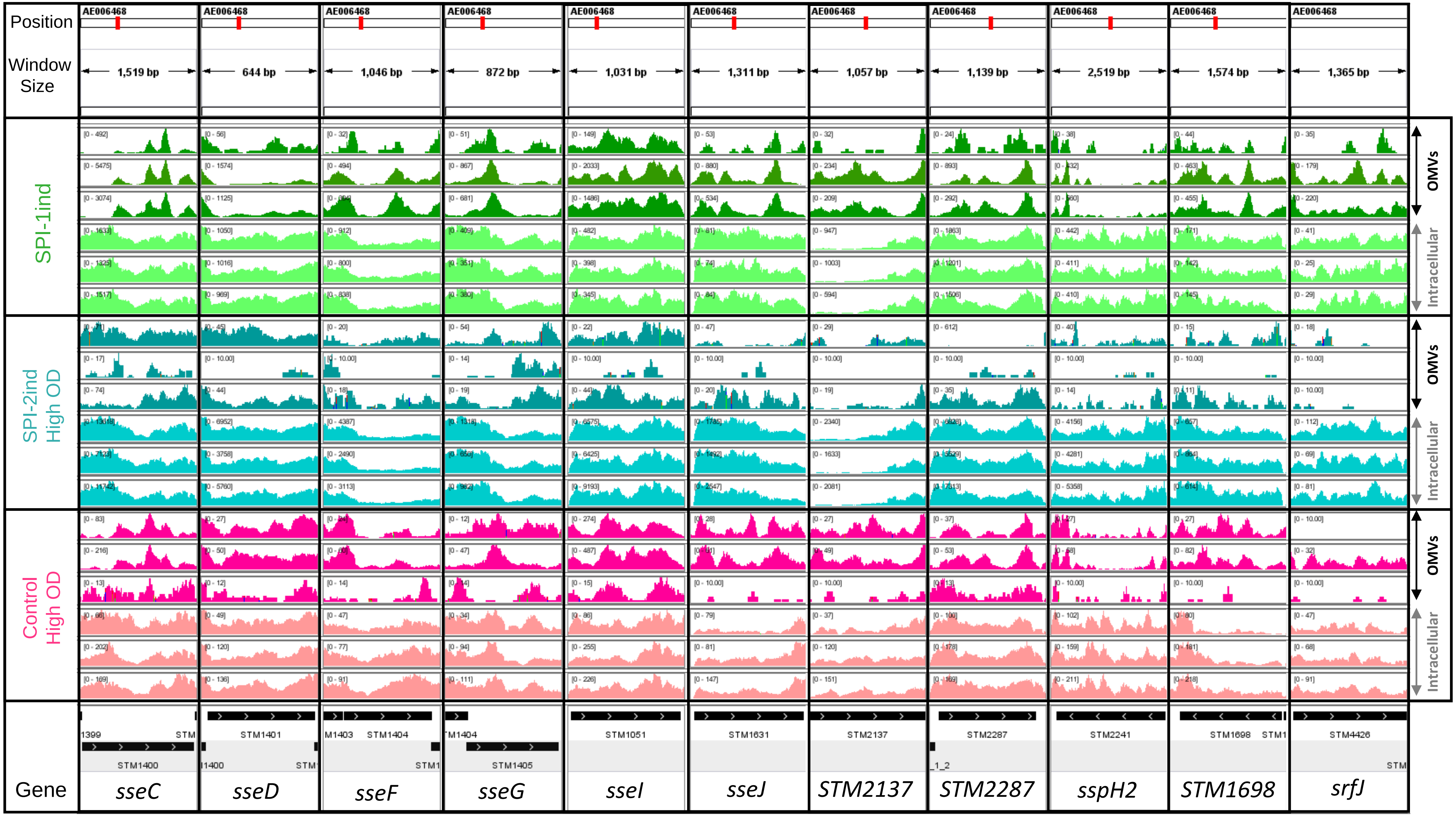
Position
Window Size
Gene
1,519 bp
644 bp
1,046 bp
872 bp
1,031 bp
1,311 bp
1,057 bp
1,139 bp
2,519 bp
1,574 bp
1,365 bp
SPI-1ind
SPI-2ind High OD
Control High OD
OMVs
Intracellular
sseC
sseD
sseF
sseG
sseI
sseJ
STM2137
STM2287
sspH2
STM1698
srfJ

Figure S11.E

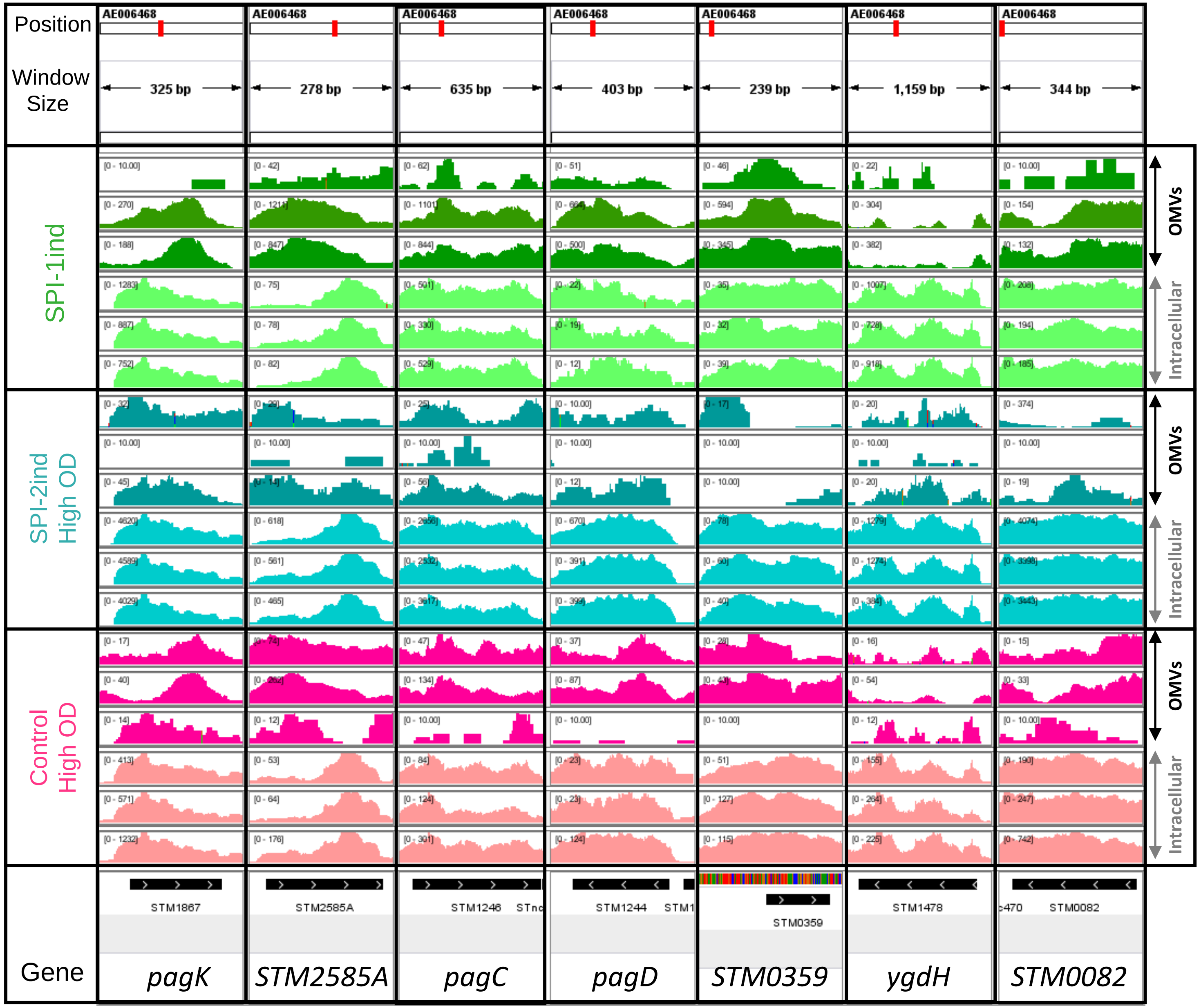

Position
Window Size
AE006468
325 bp
278 bp
635 bp
403 bp
239 bp
1,159 bp
344 bp
SPI-1ind
SPI-2ind High OD
Control High OD
OMVs
Intracellular
STM1867
STM2585A
STM1246
STM1244
STM0359
STM1478
STM0082
Gene
pagK
STM2585A
pagC
pagD
STM0359
ygdH
STM0082

Figure S11.A-E: Visualization of various intracellular and OMV-related read coverage plots of distinct known effectors in High OD culture conditions. RNA coverage from sequencing data was visualized with the Integrative Genomics Viewer software (v2.4.8) using default parameters (Robinson et al., 2011). Each plot represents the raw number of reads mapped along the observed sequence, automatically scaled relatively to the highest number of read existing in this portion of the genome. The three plots in dark colors at the top of each window represents the sequencing coverage of OMV-associated fractions, in biological triplicate for each condition. On the contrary, the three plots in lighter colors at the bottom of each window show the coverage for the same RNA but from the corresponding intracellular fractions. The position of the genes on the genome or the plasmid and the scale are precised above the charts, and their name and locus are displayed under it, according to the data extracted from the *Salmonella* LT2 genome annotation (NCBI accession number AE006468.2) or pSLT plasmid annotation (NCBI accession number AE006471.2). **A:** Effectors translocated by SPI-1 Type 3 Secretion System. **B:** Translocated by SPI-2 Type 3 Secretion System. **C-D:** Translocated by both T3SS. **E:** T3SS independent effectors.

Figure S12.A

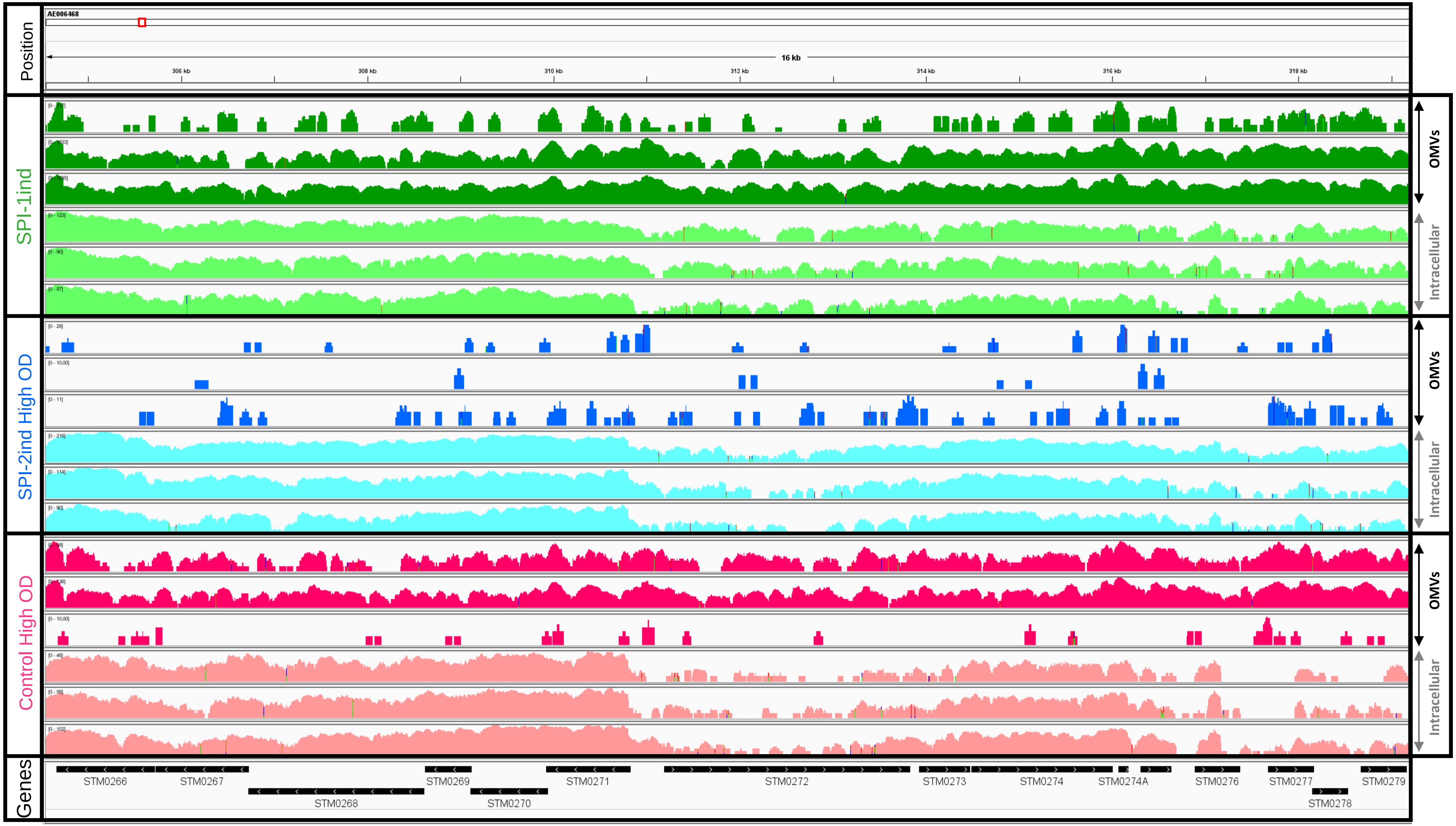

Position
AE006468
16 kb
306 kb
308 kb
310 kb
312 kb
314 kb
316 kb
318 kb
SPI-1ind
SPI-2ind High OD
Control High OD
OMVs
Intracellular
Genes
STM0266
STM0267
STM0268
STM0269
STM0270
STM0271
STM0272
STM0273
STM0274
STM0274A
STM0276
STM0277
STM0278
STM0279

Figure S12.B

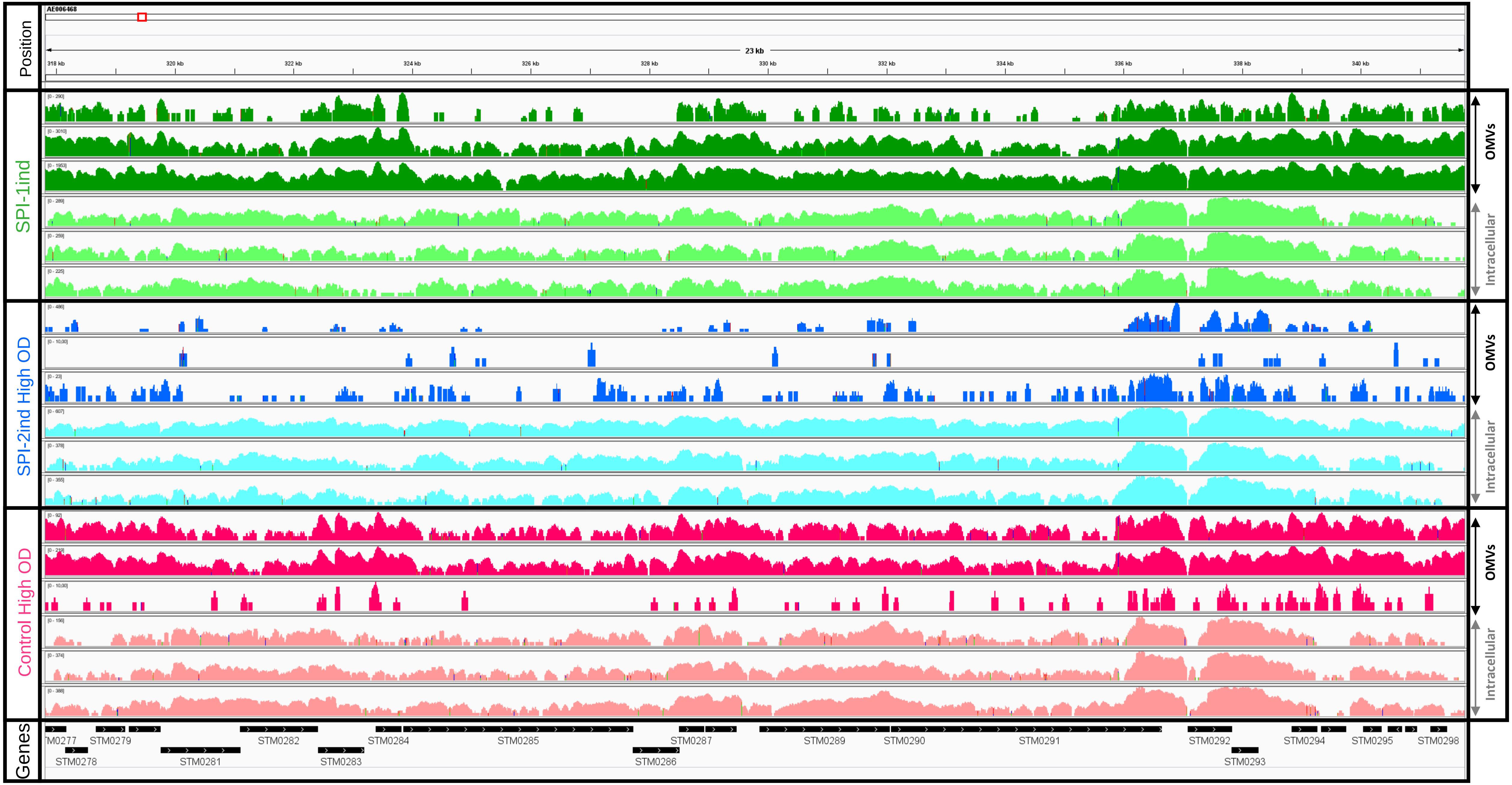

Position
AE006468
23 kb
318 kb
320 kb
322 kb
324 kb
326 kb
328 kb
330 kb
332 kb
334 kb
336 kb
338 kb
340 kb
SPI-1ind
SPI-2ind High OD
Control High OD
OMVs
Intracellular
Genes
TM0277
STM0278
STM0279
STM0281
STM0282
STM0283
STM0284
STM0285
STM0286
STM0287
STM0289
STM0290
STM0291
STM0292
STM0293
STM0294
STM0295
STM0298

Figure S12.A-B: Visualization of SPI-6 intracellular and OMV-related read coverage plots in High OD culture conditions. RNA coverage from sequencing data was visualized with the Integrative Genomics Viewer software (v2.4.8) using default parameters, but displaying the coverage in log scale (Robinson et al., 2011). Each plot represents the raw number of reads mapped along the observed sequence, automatically scaled relatively to the highest number of read existing in this portion of the genome. The three plots in dark colors at the top of each window represents the sequencing coverage of OMV-associated fractions, in biological triplicate for each condition. On the contrary, the three plots in lighter colors at the bottom of each window show the coverage for the same RNA but from the corresponding intracellular fractions. The position of the genes on the genome the scale are precised above the charts, and their locus are displayed under it, according to the data extracted from the *Salmonella* LT2 genome annotation (NCBI accession number AE006468.2) **A:** First half of the SPI-6 region. **B:** Second part of the SPI-6 region. One can note the very poor export of SPI-6-expressed RNAs in SPI-2ind High OD condition, by opposition to SPI-1ind and Control HighOD conditions where the coverage is almost complete in two of the three replicates.

Figure S13

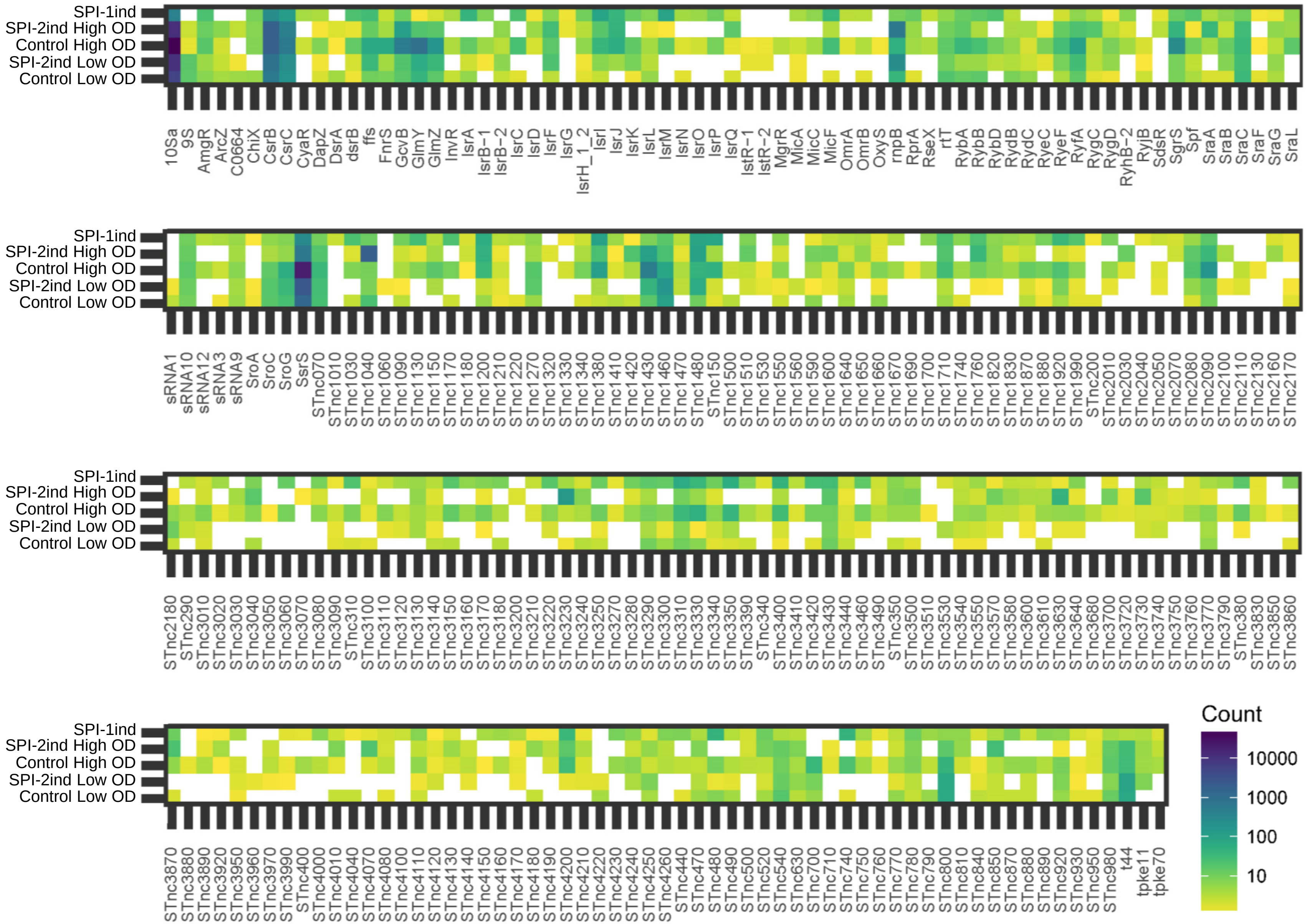
SPI-1ind
SPI-2ind High OD
Control High OD
SPI-2ind Low OD
Control Low OD
Count
10000
1000
100
10

Figure S13: Detailed list of exported sRNAs in OMVs isolated from the different culture conditions. For each OMV-associated sRNA, the corresponding mean count among triplicates of each condition is displayed as a colored square, from low (yellow) to high count (dark blue). The counts of exported sRNAs were normalized using *DESeq2*, and filtered for a minimum averaged count of one read per biological triplicate.
